# Inhibitory effect of PPARγ on NLRP3 inflammasome activation

**DOI:** 10.7150/thno.46873

**Published:** 2021-01-01

**Authors:** Ching-Chun Yang, Chih-Hsing Wu, Ta-Chun Lin, Yi-Ning Cheng, Chin-Sung Chang, Kuo-Ting Lee, Pei-Jane Tsai, Yau-Sheng Tsai

**Affiliations:** 1 Institute of Clinical Medicine, College of Medicine, National Cheng Kung University, Tainan, Taiwan, ROC.; 2 Department of Medical Laboratory Science and Biotechnology, College of Medicine, National Cheng Kung University, Tainan, Taiwan, ROC.; 3 Department of Family Medicine, National Cheng Kung University Hospital, College of Medicine, National Cheng Kung University, Tainan, Taiwan, ROC.; 4 Institute of Gerontology, College of Medicine, National Cheng Kung University, Tainan, Taiwan, ROC.; 5 Department of Surgery, National Cheng Kung University Hospital, College of Medicine, National Cheng Kung University, Tainan, Taiwan, ROC.; 6 Center for Clinical Medicine Research, National Cheng Kung University Hospital, College of Medicine, National Cheng Kung University, Tainan, Taiwan, ROC.

**Keywords:** NLRP3 inflammasome/ macrophages/ obesity/ PPARγ/ rosiglitazone

## Abstract

**Rationale:** Stimulation of the NLRP3 inflammasome by metabolic byproducts is known to result in inflammatory responses and metabolic diseases. However, how the host controls aberrant NLRP3 inflammasome activation remains unclear. PPARγ, a known regulator of energy metabolism, plays an anti-inflammatory role through the inhibition of NF-κB activation and additionally attenuates NLRP3-dependent IL-1β and IL-18 production. Therefore, we hypothesized that PPARγ serves as an endogenous modulator that attenuates NLRP3 inflammasome activation in macrophages.

**Methods:** Mouse peritoneal macrophages with exposure to a PPARγ agonist at different stages and the NLRP3 inflammasome-reconstituted system in HEK293T cells were used to investigate the additional anti-inflammatory effect of PPARγ on NLRP3 inflammasome regulation. Circulating mononuclear cells of obese patients with weight-loss surgery were used to identify the *in vivo* correlation between PPARγ and the NLRP3 inflammasome.

**Results:** Exposure to the PPARγ agonist, rosiglitazone, during the second signal of NLRP3 inflammasome activation attenuated caspase-1 and IL-1β maturation. Moreover, PPARγ interfered with NLRP3 inflammasome formation by decreasing NLRP3-ASC and NLRP3-NLRP3 interactions as well as NLRP3-dependent ASC oligomerization, which is mediated through interaction between the PPARγ DNA-binding domain and the nucleotide-binding and leucine-rich repeat domains of NLRP3. Furthermore, PPARγ was required to limit metabolic damage-associated molecular pattern-induced NLRP3 inflammasome activation in mouse macrophages. Finally, the mature caspase-1/PPARγ ratio was reduced in circulating mononuclear cells of obese patients after weight-loss surgery, which we define as an “NLRP3 accelerating index”.

**Conclusions:** These results revealed an additional anti-inflammatory role for PPARγ in suppressing NLRP3 inflammasome activation through interaction with NLRP3. Thus, our study highlights that PPARγ agonism may be a therapeutic option for targeting NLRP3-related metabolic diseases.

## Introduction

Aberrant interleukin 1β (IL-1β) production and NLR family pyrin domain containing 3 (NLRP3) inflammasome activation have been shown to elicit dysregulated inflammatory responses in several metabolic diseases, including obesity, type 2 diabetes, hyperglycemia, atherosclerosis, and non-alcoholic fatty liver disease (NAFLD) 1, 2. Excessive NLRP3 stimulation and the presence of toxic metabolic byproducts are the pathogenic hallmarks of these diseases. Increased consumption of a westernized diet, which is high in saturated fatty acids and simple sugars, results in the increased production of metabolic byproducts; these can serve as damage-associated molecular patterns (DAMPs) that initiate the innate immune response 3-5, which can include activation of the NLRP3 inflammasome 6. However, how the host attenuates NLRP3 inflammasome activation triggered by these stimuli remains poorly understood.

In macrophages, NLRP3 inflammasome activation requires two sequential signals, namely, priming and complex assembly. Priming signals, such as lipopolysaccharide (LPS), are first induced to trigger NF-κB signaling 7, 8, whereas the second signals, such as microbial toxin (nigericin), extracellular adenosine triphosphate (ATP), crystals (monosodium urate (MSU), and alum), or saturated fatty acid (palmitic acid) 6, 9 promote inflammasome assembly by triggering the interaction of components, including ASC oligomerization, NLRP3 oligomerization, and NLRP3-ASC interactions 10. Ultimately, ASC recruits and activates caspase-1 for the subsequent maturation of IL-1β and IL-18 6, 11. Structurally, NLRP3 contains a pyrin domain (PYD), a nucleotide-binding domain (NBD), and a leucine-rich repeat domain (LRR) 10, 11. During inflammasome activation, the PYD of NLRP3 interacts with ASC to initiate inflammasome assembly, whereas the NBD binds ATP to regulate NLRP3 self-oligomerization. Meanwhile, the LRR is involved in sensing stimuli and regulating protein-protein interactions during inflammasome activation 12-14. Owing to these properties, the NLRP3 domains are targets for modulation of inflammasome activation 15, 16. For example, thioredoxin interacting protein (TXNIP) 15 and NIMA-related kinase 7 (NEK7) 16 promote NLRP3 inflammasome activation via interaction with the NBD and LRR, respectively. These observations highlight that several endogenous molecules modulate NLRP3 inflammasome activation through interference with protein-protein interactions.

Peroxisome proliferator activated receptor gamma (PPARγ), a ligand-activated nuclear receptor, is a major transcriptional regulator of energy metabolism through the promotion of adipocyte differentiation as well as its insulin sensitizing potential 17. The ligand-binding domain (LBD) of PPARγ interacts with coactivators that contain two consecutive leucine-rich (LXXLL) motifs 18. Owing to the preference of the LBD for the LXXLL motif, it cannot be excluded that the LBD of PPARγ might interact with a leucine-rich moiety in the LRR of NLRP3. Although PPARγ is predominantly localized to the nucleus, where it functions as a transcription factor, it also shuttles between the nucleus and the cytoplasm 19. This suggests that PPARγ may play an important role in the cytoplasm, in addition to its canonical transcriptional activity in the nucleus.

PPARγ is known to possess anti-inflammatory activity, which is exerted through the transrepression of NF-κB and subsequent inhibition of inflammatory cytokine expression 20-22. Moreover, synthetic PPARγ agonists such as rosiglitazone and pioglitazone, clinical thiazolidinedione (TZD) drugs used in antidiabetic treatment, have been reported to attenuate IL-1β, IL-18, and caspase-1 maturation in NLRP3-associated diseases 23-26. Although rosiglitazone binds to the ligand-binding pocket located in PPARγ LBD to regulate PPARγ activity 27, it has also been shown to downregulate inflammatory responses through a PPARγ-independent mechanism 28. A recent study showed that rosiglitazone treatment decreased reactive oxygen species production, resulting in reduced NLRP3 inflammasome activation in comparative gene identification 58 (CGI-58)-deficient macrophages 29. Although PPARγ activation has been linked to anti-inflammatory effects by suppression of the NLRP3 inflammasome in neurons 30, it remains unclear whether PPARγ directly regulates the NLRP3 inflammasome. Thus, we hypothesized that PPARγ might inhibit NLRP3 inflammasome assembly/activation through multiple mechanisms, in addition to transrepression. In this study, we tested whether PPARγ directly interacts with NLRP3 to modulate NLRP3 inflammasome activation.

## Materials and Methods

### Animals

C57BL/6J and leptin-deficient (*ob/ob*) mice were obtained from National Laboratory Animal Center, Taiwan. Generation of mice carrying the modified *Pparg* locus has been described 31. *Pparg^+/+^* (WT) and *Pparg^C/-^* mice were F1 littermates from the mating of *Pparg^C/+^* mice on a C57BL/6J background with *Pparg^+/-^* mice on a 129S6 background (kindly provided by Dr. Ronald Evans at the Salk Institute) 32. Mice were bred and housed in the animal facility of National Cheng Kung University. All animal studies were performed according to protocols approved by the Institutional Animal Care and Use Committee of National Cheng Kung University.

### Mouse peritoneal macrophage isolation and treatments

For isolation of mouse peritoneal macrophages 33, *ob/ob* mice and their littermate*s* on a C57BL/6J genetic background, as well as *Pparg^C/-^* and *Pparg^+/+^* mice on a mixed C57BL/6J x 129S6 genetic background 32, were intraperitoneally injected with 3% (w/v) sterile thioglycollate 5 days prior to euthanasia. Cells were collected by lavage of the peritoneal cavity, followed with red blood cell abolishment. Cells were centrifuged and re-suspended in RPMI supplemented with 10% fetal bovine serum (FBS) and 1% penicillin and streptomycin. Mouse peritoneal macrophages were cultured at equal density of 2 × 10^6^ cells per 60-mm plate and treated with indicated compounds, including LPS (0.5 μg/mL) for 3.5 h 34, nigericin (5 μg/mL, 0.5 h), ATP (0.5 mM, 1 h) 35, palmitic acid (PA, 400 μM, 6 h) 35, MSU (200 μg/mL, 6 h) 36, alum (200 μg/mL, 6 h) 37, poly(dA:dT) (2 μg/mL, 6 h) 38, flagellin (5 μg/mL, 6 h) 39, pan caspase inhibitor (Z-VAD, 20 μM, 0.5 h) 38, caspase-1 inhibitor (YVAD, 20 μM, 0.5 h) 38, MG132 (2 h) 40, chloroquine (CQ, 2 h), and rosiglitazone (20 μM, 6.5 h) in FBS-free RPMI medium. The experimental groups in peritoneal macrophages are: untreated control, LPS priming, LPS priming plus nigericin (or other Signal-2 activators) for induction of NLRP3 inflammasome activation, and inflammasome activation combined with rosiglitazone (or other indicated compounds).

### HEK293T cell transfection and treatments

HEK293T cells with the passage from 7 to 17 were maintained in DMEM supplemented with 10% FBS, and cultured at equal density of 2 × 10^6^ cells per 60-mm plate for indicated experiments. Cells were starved in DMEM for 24 h prior to transfection of indicated inflammasome components (a gift from Dr. Ming-Zong Lai, Academia Sinica, Taiwan) 41 and PPARγ plasmids, including NLRP3 and PPARγ constructed mutants (Table S1), by TurboFect™ Transfection Reagent (ThermoFisher Scientific, Waltham, MA, USA) or lipofectamine 3000 Reagent (Invitrogen, Carlsbad, CA, USA). pcDNA4 and pCMV2 plasmids were used as the transfection controls of inflammasome components and PPARγ plasmids, respectively. For ASC oligomerization, cells were transfected with ASC (100 ng), HA-NLRP3 (100 ng), and PPARγ (200 ng) for 24 h. Resultant pellets from cells were collected and analyzed by immunoblotting. For immunoprecipitation, cells were transfected with HA-NLRP3 (100 ng), ASC (100 ng), myc-NLRP3 (100 ng) and PPARγ (200 ng) for 24 h. Cell lysates were collected for immunoprecipitation assay. For IL-1β maturation, cells were transfected with HA-NLRP3 (20 ng), caspase-1 p45 (50 ng), pro-IL-1β (100 ng), ASC (20 ng), and PPARγ (1 μg) for 24 h. Transfection dosage of PPARγ, as well as the cell viability, was tested in HEK293T cells (Figure S1A-B). Cells were treated with rosiglitazone for 24 h after transfection. Culture medium was collected for IL-1β detection and cell lysates were collected for indicated plasmid protein expression by immunoblotting. The experimental groups in HEK293T cells are: non-transfected control, inflammasome component transfection, and inflammasome components co-transfected with PPARγ.

### Immunoblotting

For immunoblotting, the equal amounts of total proteins were subjected to SDS-PAGE, transferred to PVDF membranes, and incubated with primary antibodies (Table S2) followed by horseradish peroxidase (HRP)-conjugated secondary antibodies (Vector Laboratories, Burlingame CA, USA) (Table S2). Immunoreactive protein detection was performed with an enhanced chemiluminescence detection system (GE Healthcare, Pittsburgh, PA, USA).

For ASC oligomerization assay, resultant pellets from cells were washed with cold 1X PBS, crosslinked with 4 mM disuccinimidyl suberate (DSS, Sigma-Aldrich, St. Louis, MO, USA) for 30 min, and pelleted by centrifugation. The crosslinked pellets were resuspended by the sample buffer for immunoblotting analysis.

### Immunoprecipitation

Immunoprecipitation was performed in the total lysate of mouse peritoneal macrophages or HEK293T cells using a Dynabeads protein G kit (Novex, ThermoFisher Scientific, Waltham, MA, USA). Primary and IgG control (1 μg, Table S2) antibodies were incubated with total protein lysate (1 mg) overnight at 4 °C, and then incubated with Dynabeads for 10 min at RT. After washing with PBST buffer (0.1% Tween-20) and heating with 70 ºC for 10 min, the tube was placed on the magnet and the supernatant was analyzed by immunoblotting.

### Immunofluorescence

Cells were fixed by 4% paraformaldehyde, permeabilized by 0.5% Triton X-100, blocked with 3% BSA, and incubated with indicated primary antibodies (Table S2) in 3% BSA overnight at 4 °C. After washing with cold 1X PBS, samples were incubated with the secondary antibodies for 1 h at RT, and then mounted in Fluoroshield with DAPI of mounting media (ImmunoBioScience, Mukilteo, Washington, USA). The images were visualized by confocal microscopy (C1-Si, Nikon, Tokyo, Japan) with a 60× oil objective lens. Quantification of positive signals and the colocalization coefficient of all images were analyzed by ImageJ software.

### Proximity ligation assay (PLA) *in situ*

Cells were fixed by 4% paraformaldehyde, permeabilized by 0.5% Triton X-100, and processed for Duolink® PLA *in situ* assay kit (Sigma-Aldrich, St. Louis, MO, USA). The images were visualized by confocal microscopy (C1-Si, Nikon, Tokyo, Japan) with a 60× oil objective lens. Quantification of positive signals was measured by ImageJ software.

### Human and peripheral blood mononuclear cells (PBMCs) procurement

Patients who attended the Weight Control Clinic in National Cheng Kung University Hospital, Tainan, Taiwan for bariatric surgery (mini-gastric bypass, sleeve gastrectomy, and gastric banding; BMI ≥ 32 kg/m^2^), as well as control volunteers with BMI lower than 35 and without diagnosis of metabolic syndrome, were included in our study. Information of subjects included was provided in Table S3. Blood samples were collected before and six or twelve months after surgery. Peripheral blood mononuclear cells (PBMCs) were isolated from obese subjects undergoing bariatric surgery by density gradient centrifugation using Ficoll-Paque™ PREMIUM (GE Healthcare, Pittsburgh, PA, USA) 42. Homeostatic model assessment (HOMA) index was calculated as the result of fasting glucose and insulin level divided by 22.5. All the informed consent, blood collection procedure, clinical data acquisition and postoperative report of adverse effect were approved and regulated by the Institutional Review Board of National Cheng Kung University Hospital.

### IL-1β enzyme-linked immunosorbent assay (ELISA)

IL-1β level in the culture medium or total cell lysates of human PBMCs were measured using mouse or human IL-1β ELISA kits (eBioscience, ThermoFisher Scientific, Waltham, MA, USA).

### Data analysis

Values are presented as mean ± SEM. Statistical analyses were executed by Student's *t*-test, or one-way and two-way ANOVA followed by Fisher's least significant difference test. The analyses for the results in human PBMCs were executed by paired *t*-test and Spearman's rank correlation coefficients. Statistically significance was set at *P* value < 0.05.

## Results

### Rosiglitazone attenuated NLRP3 inflammasome activation

**Figure 1:**
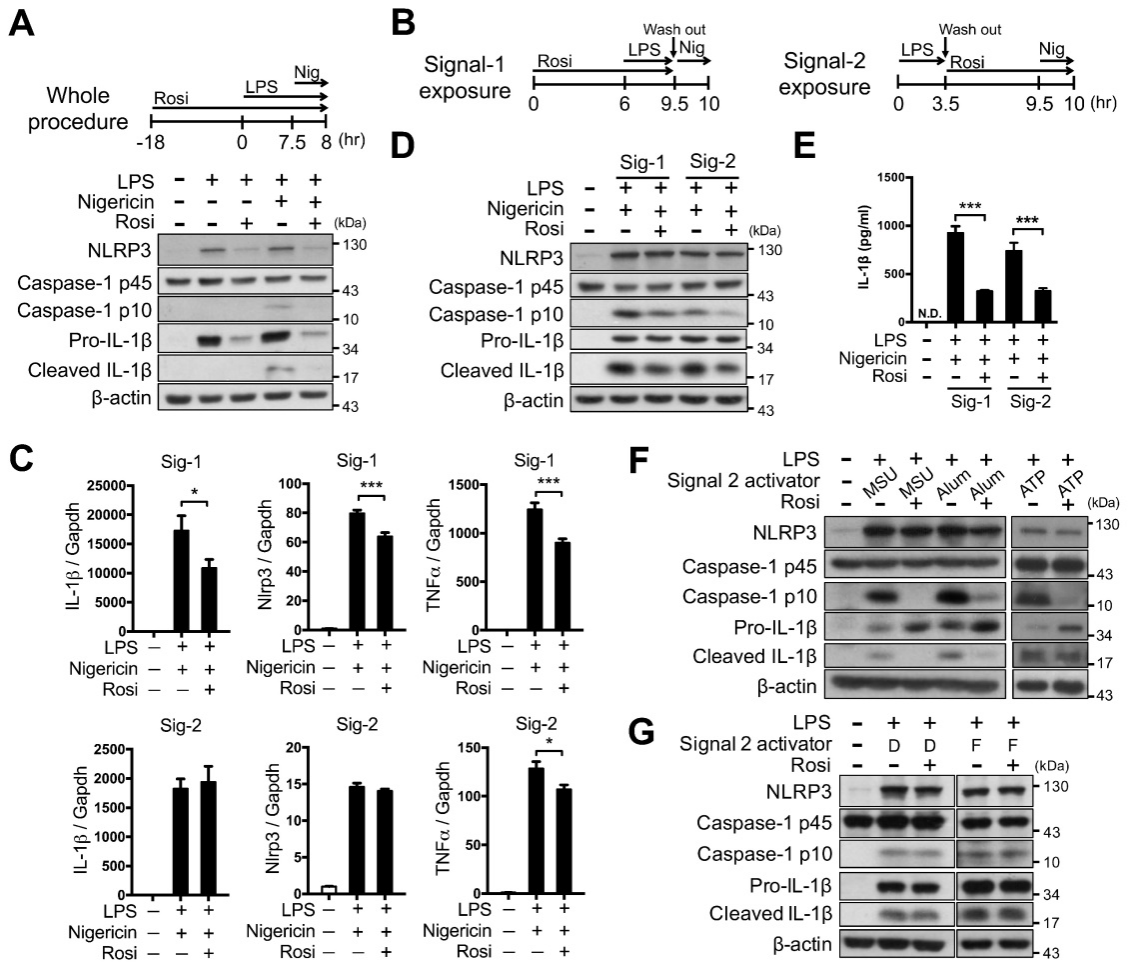
** Rosiglitazone attenuated NLRP3 inflammasome activation.** (**A-E**) Immunoblot analysis of caspase-1 activation and IL-1β maturation in mouse peritoneal macrophages. Schematic diagrams show the experimental design of co-treatment with Rosi in (**A**) the whole procedure, or (**B**) the signal 1 (Signal-1 exposure) and signal 2 (Signal-2 exposure) of NLRP3 inflammasome activation. **(C)** Expression of NLRP3, IL-1β, and TNFα was detected in Signal-1 (upper panels) and Signal-2 (lower panels) exposure protocols from four independent experiments. mRNA levels are expressed relative to average expression in the unstimulated control group. (**D**) Immunoblot analysis of caspase-1 activation and IL-1β maturation in mouse peritoneal macrophages treated with rosiglitazone by Signal-1 and Signal-2 exposure protocols. (**E**) IL-1β level was detected by ELISA from six independent experiments. (**F-G**) Immunoblot analysis of caspase-1 activation and IL-1β maturation in LPS-primed mouse peritoneal macrophages treated with (**F**) MSU, alum, and ATP, or with (**G**) dsDNA (D) and flagellin (F) with the Signal-2 exposure protocol. Caspase-1 p10 and mature IL-1β are collected from culture supernatants and others are from cell lysates. *P < 0.05 and ***P < 0.001 by one-way ANOVA with Fisher's LSD test. Representative blots in (**A**) and (**D**) are from three independent experiments; and in (**F**) and (**G**) are from one and two independent experiments, respectively. Experiment replicates are shown in Figure S10.

We first investigated whether PPARγ activation could modulate NLRP3 inflammasome activation. Rosiglitazone treatment throughout the entire period of NLRP3 inflammasome activation led to a substantial decrease in NLRP3-dependent caspase-1 activation and IL-1β maturation in mouse peritoneal macrophages, and also reduced the levels of NLRP3 and pro-IL-1β (Figure 1A). To further determine how PPARγ activation affected the first and second signals of NLRP3 inflammasome activation, we employed Signal-1 and Signal-2 exposure protocols, as shown in Figure 1B. The Signal-1 exposure protocol affected the expression of NLRP3, IL-1β, and TNFα, whereas the Signal-2 exposure protocol did not elicit significant differences in the expression of IL-1β and NLRP3 (Figure 1C). Signal-1 and Signal-2 exposure treatments both led to a decrease in the levels of mature caspase-1 and IL-1β (Figure 1D-E), suggesting that PPARγ activation can attenuate the second signal for NLRP3 inflammasome activation. Moreover, Signal-2 exposure treatment also led to a reduction in the levels of mature caspase-1 and IL-1β that were induced by MSU, alum, and ATP, known second-signal activators of the NLRP3 inflammasome (Figure 1F). However, Signal-2 exposure treatment did not affect the levels of mature caspase-1 and IL-1β that were induced by absent in melanoma 2 (AIM2; recognizes cytosolic dsDNA, poly(dA:dT)) or NLR family CARD domain containing 4 (NLRC4 inflammasome, activated by bacterial *flagellin*) (Figure 1G). These results suggested that the regulation of PPARγ in the second signal of inflammasome activation is specific to the NLRP3 inflammasome.

### PPARγ interfered with NLRP3-NLRP3 and NLRP3-ASC interactions

**Figure 2:**
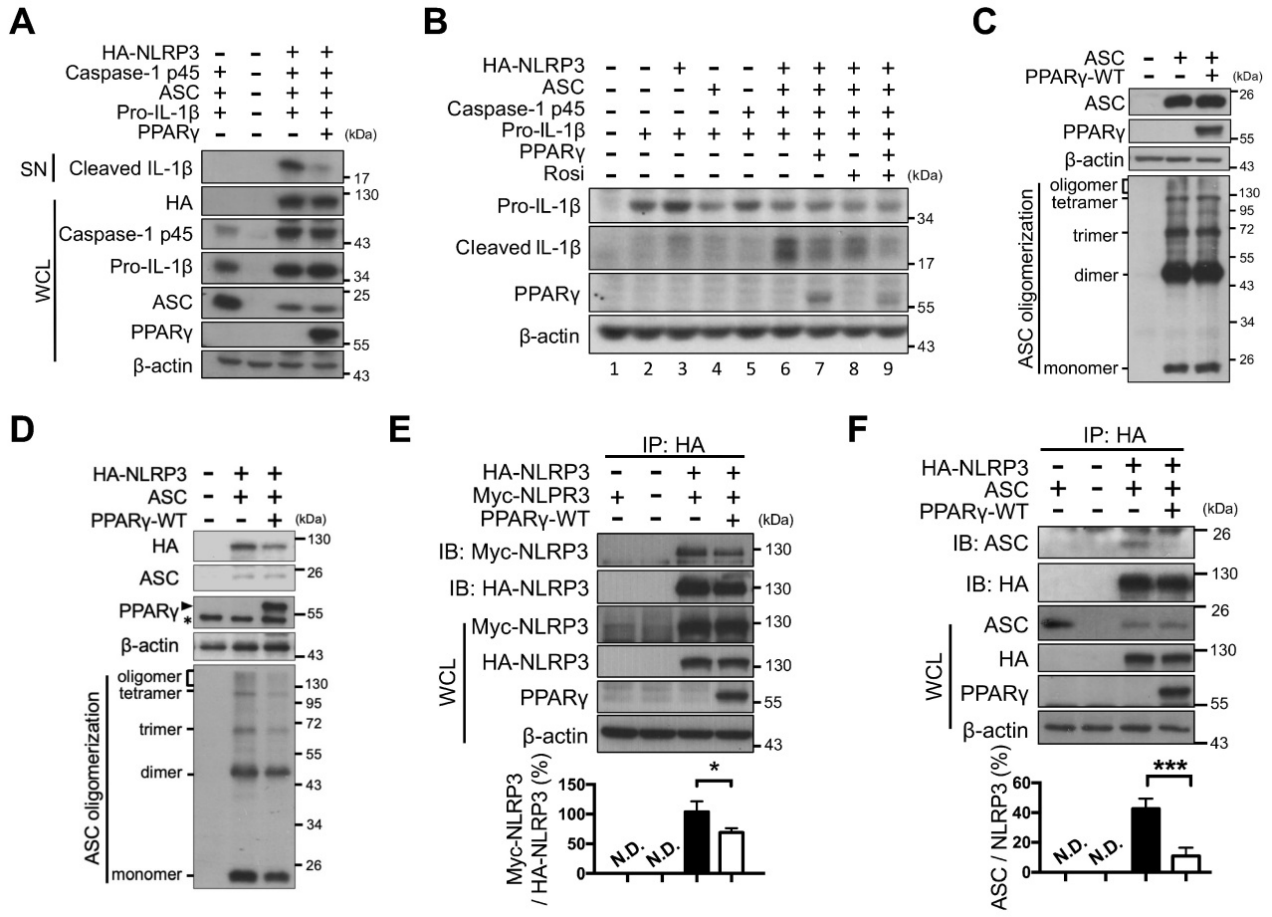
** PPARγ interfered NLRP3 oligomerization and NLRP3-ASC interaction.** (**A**) Immunoblot analysis of mature IL-1β in the supernatant and indicated components and PPARγ in the cell lysates of NLRP3 inflammasome-reconstituted HEK293T cells transfected with indicated components. (**B**) Immunoblot analysis of mature IL-1β in the supernatant, and pro-IL-1β and PPARγ in the cell lysates of NLRP3 inflammasome-reconstituted HEK293T cells transfected with indicated components. Rosiglitazone (Rosi, 20μM) was treated for 24 h after transfection. (**C-D**) Immunoblot analysis of ASC oligomerization in HEK293T cells transfected with ASC, NLRP3 and PPARγ. PPARγ band is labeled with arrowhead and a non-specific band is labeled with asterisk in (**D**). (**E**) Immunoprecipitation and immunoblot analysis of the interaction between Myc-tagged NLRP3 and HA-tagged NLRP3 in HEK293T cells. Quantification of Myc-tagged NLRP3 relative to the level of HA-tagged NLRP3 from four independent experiments. (**F**) Immunoprecipitation and immunoblot analysis of the interaction between HA-tagged NLRP3 and ASC in HEK293T cells. Quantification of ASC relative to the level of HA-tagged NLRP3 from three independent experiments. *P < 0.05 and ***P < 0.001 by one-way ANOVA with Fisher's LSD test in (**E-F**). Representative blots are from three independent experiments. Experiment replicates are shown in Figure S11.

Next, NLRP3 inflammasome components (NLRP3, ASC, caspase-1, and pro-IL-1β) were reconstituted and artificially expressed in HEK293T cells under the control of the constitutive CMV promoter (no endogenous expression; Figure 2A). Combined expression of exogenous HA-tagged NLRP3, pro-caspase-1, ASC, and pro-IL-1β induced the secretion of mature IL-1β (Figure 2A, lane 6 of Figure 2B). However, PPARγ overexpression (Figure 2A, lane 7 of Figure 2B and Figure S1A) or rosiglitazone treatment alone (lane 8 of Figure 2B) led to decreased secretion of mature IL-1β, whereas a combination of both further reduced IL-1β secretion (lane 9 of Figure 2B). These results suggested that both the overexpression and activation of PPARγ can inhibit NLRP3 inflammasome activation.

We then examined whether PPARγ exerted its inhibitory effect on NLRP3 inflammasome activation by interfering with NLRP3 complex formation. NLRP3 inflammasome complex formation is known to require ASC oligomerization as well as NLRP3-NLRP3 and NLRP3-ASC interactions 10. Overexpression of ASC in HEK293T cells resulted in ASC oligomerization, but co-expression with PPARγ did not affect ASC oligomerization (Figure 2C). However, in the presence of NLRP3, oligomerization of ASC was attenuated by PPARγ co-expression (Figure 2D). We also used co-immunoprecipitation in HEK293T cells to examine whether PPARγ affected NLRP3-NLRP3 interaction. We found that HA-tagged NLRP3 co-immunoprecipitated with Myc-tagged NLRP3, but this was attenuated by PPARγ co-expression (Figure 2E). In addition, HA-tagged NLRP3 co-immunoprecipitated with ASC, but this was also attenuated by PPARγ co-expression (Figure 2F). Combined, these results suggest that the inhibitory effect of PPARγ on NLRP3 inflammasome activation is likely to be exerted through the inhibition of NLRP3-NLRP3, NLRP3-ASC interactions, and NLRP3-depenent ASC oligomerization.

### PPARγ interacted with NLRP3

**Figure 3:**
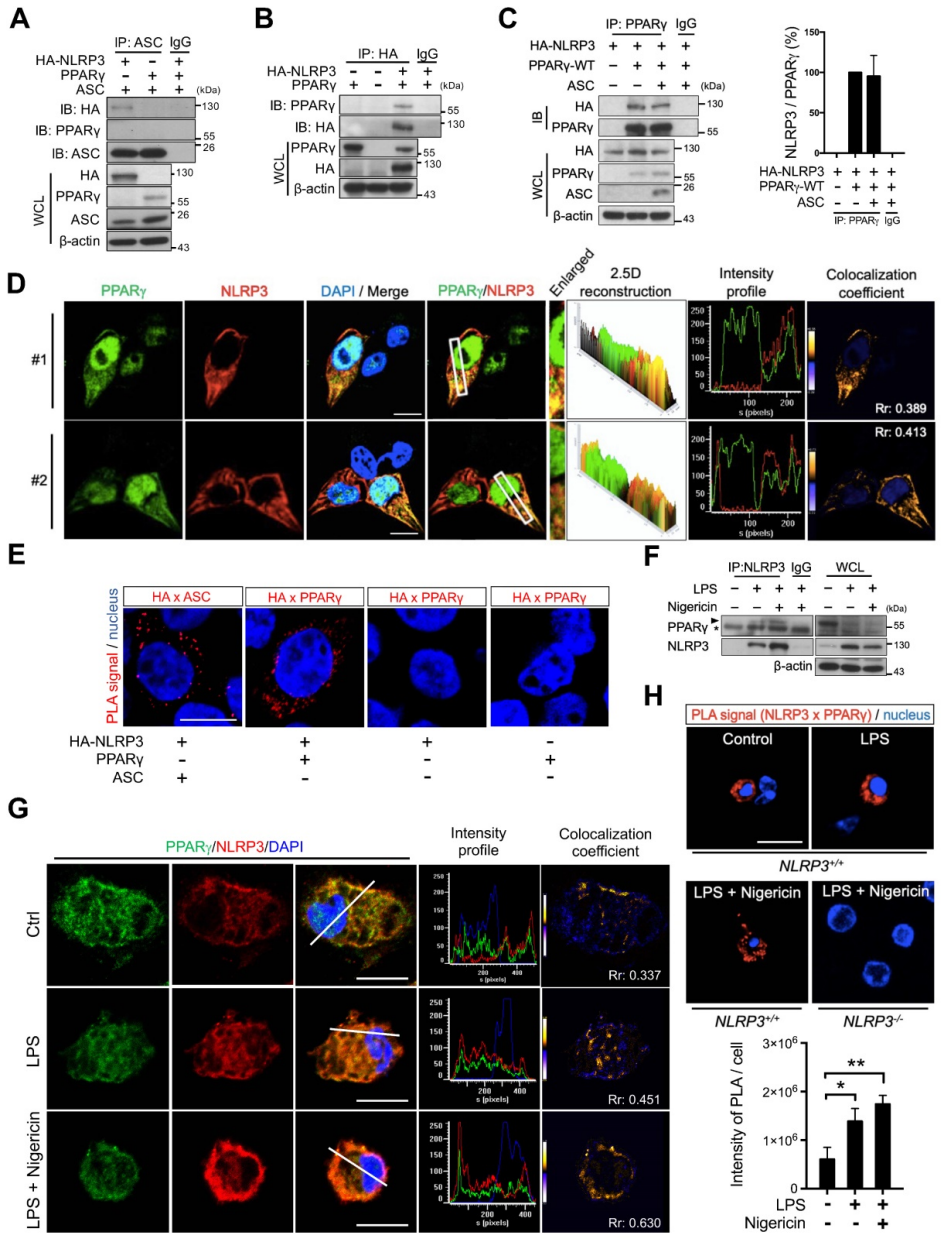
** PPARγ interacted with NLRP3 during NLRP3 inflammasome activation.** (**A**) Immunoprecipitation and immunoblot analysis of the interaction between HA-tagged NLRP3, ASC, and PPARγ in HEK293T cells. (**B**) Immunoprecipitation and immunoblot analysis of the interaction between HA-tagged NLRP3 and PPARγ in HEK293T cells. (**C**) Immunoprecipitation and immunoblot analysis of the interaction between HA-tagged NLRP3 and PPARγ in presence of ASC in HEK293T cells. Quantification of HA-tagged NLRP3 relative to the level of PPARγ from three independent experiments. (**D**) Confocal images and co-localization analysis of HA-tagged NLRP3 (*red*) and PPARγ (*green*) in HEK293T cells. The enlarged images highlight the representative colocalization with 4× magnification from white squares in the overlay images. The 2.5-dimensional reconstruction analysis of the respective enlarged images is shown. The fluorescence intensity profile from green and red channels is shown. The colocalization coefficient is presented with yellow or blue color pixels indicate colocalization or segregation, respectively. Scale bar, 10 µm. (**E**) *In situ* proximity ligation assay (PLA) images for interaction between HA-tagged NLRP3 and PPARγ in HEK293T cells. Interaction between HA-tagged NLRP3 and ASC is performed as a positive control. The positive signals are represented by the fluorescent red dots. Scale bar, 10 µm. (**F**) Immunoprecipitation and immunoblot analysis of the interaction between NLRP3 and PPARγ in mouse peritoneal macrophages. PPARγ band is labeled with arrowhead and a non-specific band is labeled with asterisk. (**G**) Confocal images of NLRP3 (*red*) and PPARγ (*green*) in mouse peritoneal macrophages. The fluorescence intensity profile from green, red, and blue channels is shown. The colocalization coefficient is presented with yellow or blue color pixels for colocalization or segregation, respectively. Scale bar, 10 µm. (**H**) PLA images for interaction between NLRP3 and PPARγ in mouse peritoneal macrophages. Scale bar, 10 µm. The peritoneal macrophages from* Nlrp3^-/-^* mice are used as a control. Quantification of PLA signal per cell is presented. Pearson's coefficient (Rr) of the colocalization is shown in (**D and G**). *P < 0.05 and **P < 0.01 by one-way ANOVA with Fisher's LSD test. OE, overexpression; WCL, whole cell lysate; SN, supernatant. Representative blots in (**B**), (**C**), and (**F**) are from three independent experiments; and in (**A**) are from two independent experiments. Experiment replicates are shown in Figure S12.

To investigate whether PPARγ exerted its inhibitory effect on NLRP3 inflammasome formation by directly interacting with NLRP3, we expressed HA-tagged NLRP3, ASC, and PPARγ in HEK293T cells and analyzed their interaction using co-immunoprecipitation. ASC co-immunoprecipitated with HA-tagged NLRP3 but not with PPARγ (Figure 3A), whereas HA-tagged NLRP3 co-immunoprecipitated with PPARγ (Figure 3B). Moreover, co-expression with ASC did not affect the interaction between PPARγ and NLRP3 (Figure 3C). Immunofluorescence staining and a proximity ligation assay (PLA) further confirmed that NLRP3 and PPARγ colocalized in the cytosol of HEK293T cells (Figure 3D-E). We also examined the NLRP3-PPARγ interaction in mouse peritoneal macrophages. LPS priming and nigericin co-treatment resulted in increased interaction between NLRP3 and PPARγ (Figure 3F). Reverse co-immunoprecipitation also confirmed that PPARγ co-immunoprecipitated with NLRP3 in untreated control, LPS priming, and nigericin co-treatment macrophages (Figure S2). Furthermore, NLRP3 and PPARγ were observed to colocalize in the cytosol of untreated, control macrophages, and LPS treatment and nigericin co-treatment increased this colocalization (Figure 3G). Interestingly, the PPARγ signal was also observed in NLRP3 aggregates in the nigericin-treated group in spite of a decreased PPARγ signal (Figure 3G and S3A). Consistent with these observations, the PLA also showed that NLRP3 and PPARγ interacted in untreated, control macrophages, and treatment with LPS and nigericin increased the colocalization signal intensity (Figure 3H). These results suggest that the interaction between NLRP3 and PPARγ in the cytosol occurs in the untreated stage, and that NLRP3 inflammasome activation increases this interaction.

### PPARγ DNA-binding domain mediated the interaction with NLRP3

**Figure 4:**
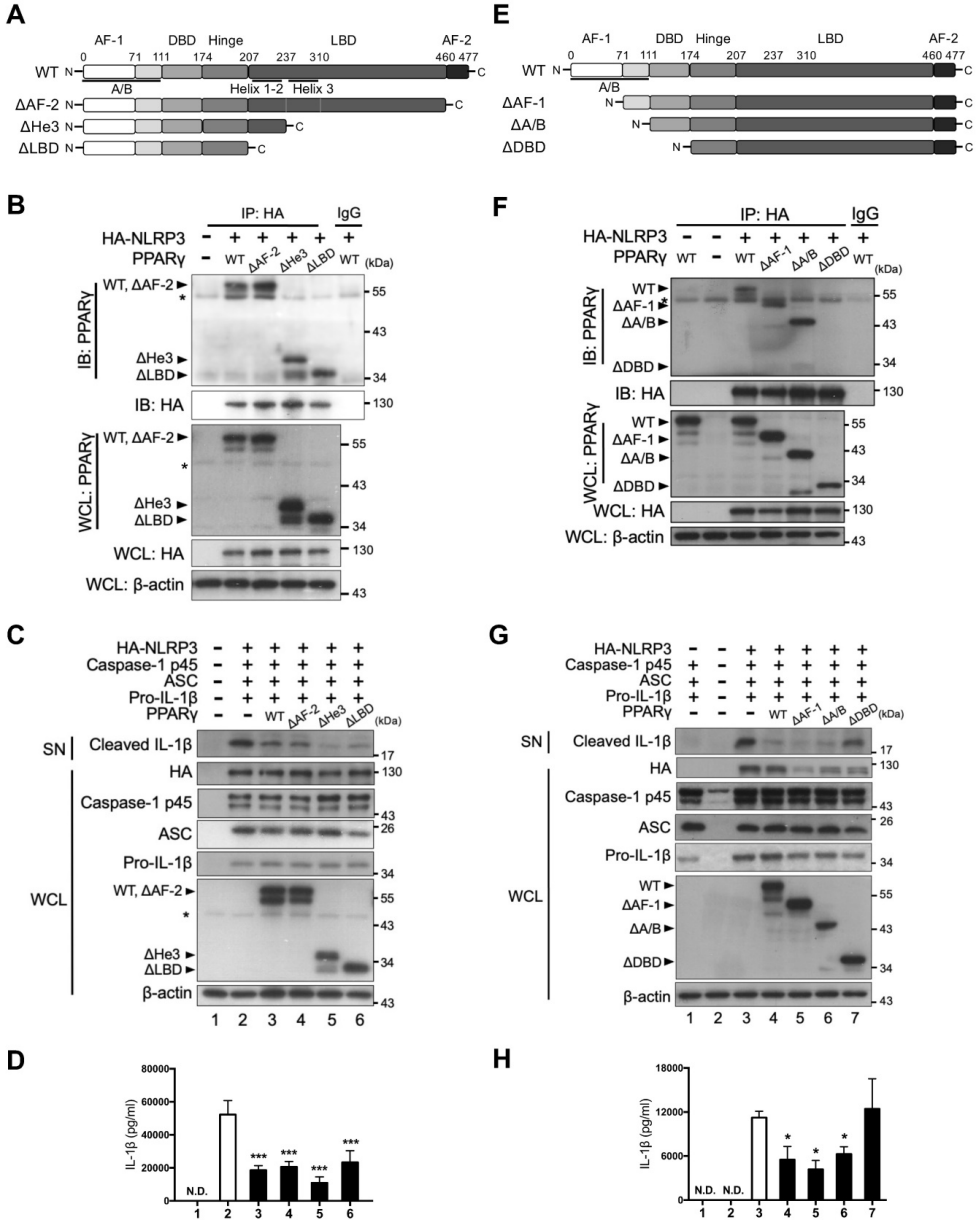
** PPARγ DBD mediated the interaction with NLRP3 and the inhibitory effect on NLRP3 inflammasome activation.** (**A**) Schematic diagrams show PPARγ LBD-truncated mutants (ΔAF-2, ΔHe3, and ΔLBD). (**B**) Immunoprecipitation and immunoblot analysis of the interaction between LBD-truncated PPARγ and HA-tagged NLRP3 in HEK293T cells. (**C**) Immunoblot analysis of mature IL-1β in the supernatant (SN) and NLRP3 inflammasome components in whole cell lysate (WCL) of NLRP3 inflammasome-reconstituted HEK293T cells overexpressing LBD-truncated PPARγ. (**D**) IL-1β levels detected by ELISA from three independent experiments. (**E**) Schematic diagrams show PPARγ N-terminal truncated mutants (ΔAF-1, ΔA/B, and ΔDBD). (**F**) Immunoprecipitation and immunoblot analysis of the interaction between N-terminal truncated PPARγ and HA-tagged NLRP3 in HEK293T cells. (**G**) Immunoblot analysis of mature IL-1β in the SN and NLRP3 inflammasome components in WCL of NLRP3 inflammasome-reconstituted HEK293T cells overexpressing N-terminal truncated PPARγ. (**H**) IL-1β levels detected by ELISA from three independent experiments. AF-2, activation function 2; He3, helix-3; LBD, Ligand binding domain; AF-1, activation function 1; DBD, DNA binding domain. PPARγ band is labeled with arrowhead and a non-specific band is labeled with asterisk. *P < 0.05 and ***P < 0.001 by one-way ANOVA with Fisher's LSD test. Representative blots are from three independent experiments. Experiment replicates are shown in Figure S13.

We further assessed which domain of PPARγ was involved in its interaction with NLRP3. Because we found that both the NBD and LRR of NLRP3 contain four LXXLL motifs, we speculated that the interaction between NLRP3 and PPARγ could be mediated through these motifs and the LBD of PPARγ. To test this, we first constructed three LBD-truncated PPARγ variants, including deletion of the AF-2 domain (ΔAF-2), helix-3 (ΔHe3), or complete LBD (ΔLBD) (Figure 4A), and examined their interaction with NLRP3 in HEK293T cells. HA-tagged NLRP3 co-immunoprecipitated not only with full-length (wild-type [WT]) PPARγ, but also with the three LBD-truncated forms of this protein (ΔAF-2, ΔHe3, and ΔLBD; Figure 4B). Moreover, the three LBD-truncated forms of PPARγ retained the ability to inhibit the secretion of mature IL-1β, as evidenced by the western blot and ELISA results (Figure 4C-D). These data indicate that the interaction between NLRP3 and PPARγ is not mediated through the LBD of PPARγ.

We also constructed three N-terminal-truncated PPARγ variants, with deletion of the AF-1 domain (ΔAF-1), the entire A/B domain (ΔA/B), or the entire A/B and DNA-binding domains (ΔDBD) (Figure 4E). Interestingly, HA-tagged NLRP3 co-immunoprecipitated with the ΔAF-1 and ΔA/B forms of PPARγ, but not with the ΔDBD form (Figure 4F). Consistent with this observation, the ΔAF-1 and ΔA/B forms of PPARγ retained the ability to inhibit the secretion of mature IL-1β, whereas ΔDBD did not (Figure 4G-H). These results suggest that the DBD of PPARγ mediates its interaction with NLRP3.

### Nucleotide-binding and leucine-rich repeat domains of NLRP3 are involved in its interaction with PPARγ

**Figure 5:**
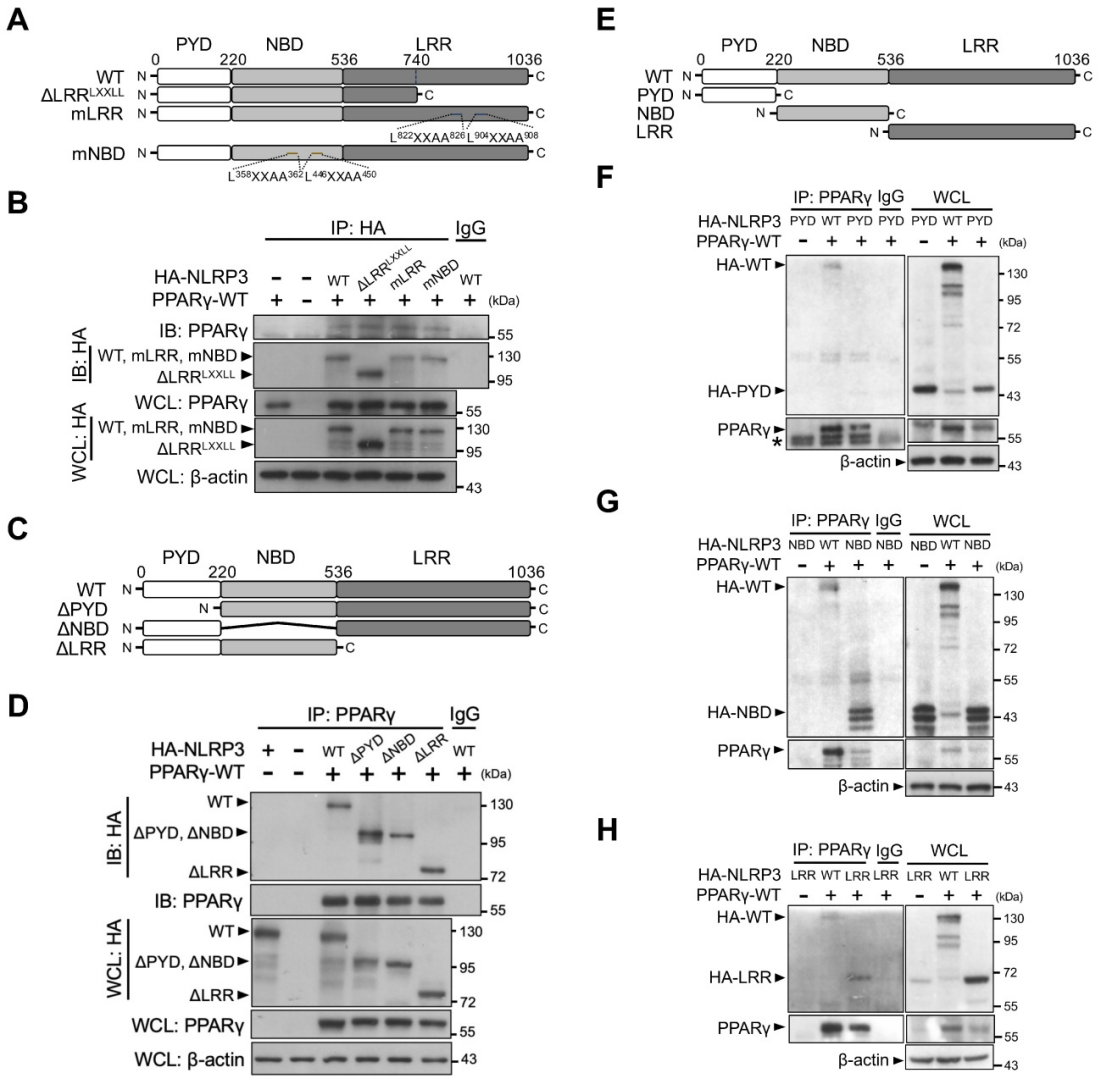
** NLRP3 NBD and LRR domains are involved in NLRP3-PPARγ interaction.** (**A**) Schematic diagrams show NLRP3 LXXLL mutants (ΔLRR^LXXLL^, mLRR, and mNBD). (**B**) Immunoprecipitation and immunoblot analysis of the interaction between NLRP3 LXXLL mutants and PPARγ in HEK293T cells. (**C**) Schematic diagrams show NLRP3 truncated mutants (ΔPYD, ΔNBD, and ΔLRR). (**D**) Immunoprecipitation and immunoblot analysis of the interaction between NLRP3 truncated mutants and PPARγ in HEK293T cells. (**E**) Schematic diagrams show NLRP3 truncated deletions to retain only PYD, NBD, and LRR. (**F-H**) Immunoprecipitation and immunoblot analysis of the interaction between NLRP3 truncated deletions and PPARγ in HEK293T cells. LRR, leucine-rich repeat domain; NBD, nucleotide-binding domain; PYD, pyrin domain. PPARγ and truncated NLRP3 bands are labeled with arrowhead. Representative blots in (**D**), (**F**), (**G**), and (**H**) are from three independent experiments; and in (**B**) are from two independent experiments. Experiment replicates are shown in Figure S14.

To elucidate which NLRP3 domain is involved in its interaction with PPARγ, we first constructed two LXXLL-mutated forms of NLRP3 (two mutated LXXLL motifs within the NBD [mNBD] and two within the LRR [mLRR]) as well as an LRR-truncated variant of NLRP3 that resulted in the deletion of two LXXLL motifs (ΔLRR^LXXLL^) (Figure 5A). All HA-tagged forms of NLRP3 (full-length [WT], mNBD, mLRR, and ΔLRR^LXXLL^) co-immunoprecipitated with PPARγ in HEK293T cells (Figure 5B). We then created three truncated variants of NLRP3, with the deletion of PYD (ΔPYD), NBD (ΔNBD), or LRR (ΔLRR) (Figure 5C). PPARγ co-immunoprecipitated with these three truncated forms of NLRP3 (ΔPYD, ΔNBD, and ΔLRR) (Figure 5D). These results suggest that interaction between NLRP3 and PPARγ may involve more than one domain within NLRP3. To test this, we further designed truncated forms of NLRP3 that retained only PYD, NBD, or LRR (Figure 5E). PPARγ co-immunoprecipitated with the NBD and LRR, but not with PYD (Figure 5F-H). These results indicated that both the NBD and LRR of NLRP3 are involved in its interaction with PPARγ interaction.

### PPARγ is downregulated during NLRP3 inflammasome activation

**Figure 6:**
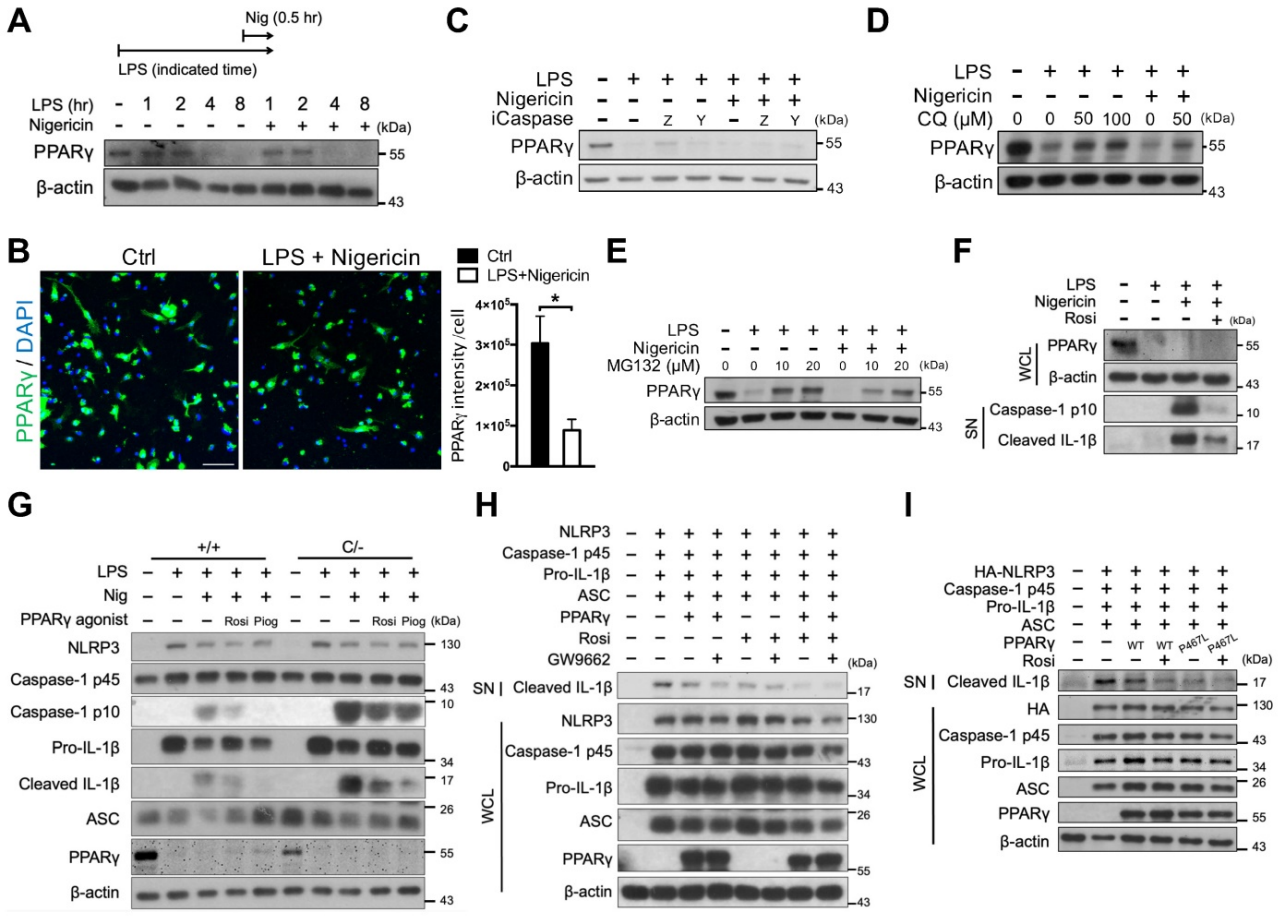
** Rosiglitazone attenuated NLRP3 inflammasome activation in a PPARγ-independent mechanism.** (**A**) Immunoblot analysis of PPARγ in mouse peritoneal macrophages treated with LPS and nigericin for indicated time. A schematic diagram on the top shows the experimental design of LPS and nigericin treatment. (**B**) Immunofluorescent staining and quantification of PPARγ (*green*) intensity per cell in mouse peritoneal macrophages (n = 5 in each group). Scale bar, 50 µm. Negative controls are shown in Figure S4. (**C-E**) Immunoblot analysis of PPARγ in mouse peritoneal macrophages treated with (**C**) caspase-1 inhibitors (iCaspase; ZVAD (Z, 20 μM) and YVAD (Y, 20 μM)), (**D**) chloroquine (CQ), and (**E**) MG132. (**F**) Immunoblot analysis of PPARγ in whole cell lysate and caspase-1 activation and mature IL-1β in the supernatant in mouse peritoneal macrophages treated with rosiglitazone. (**G**) Immunoblot analysis of caspase-1 activation and IL-1β maturation in wild-type (*Pparg^+/+^*) and *Pparg^C/-^* mouse peritoneal macrophages treated with rosiglitazone (Rosi, 20 μM) or pioglitazone (Piog, 20 μM) with Signal-2 exposure protocol. (**H-I**) Immunoblot analysis of mature IL-1β in the supernatant and indicated components and PPARγ in the cell lysates of NLRP3 inflammasome-reconstituted HEK293T cells transfected with indicated components and PPARγ (WT and P467L mutant). Rosiglitazone (Rosi, 20 μM) and GW9662 (20 μM) were treated for 24 h after transfection. *P < 0.05 by Student's *t*-test. WCL, whole cell lysate; SN, supernatant. Representative blots in (**A**), (**E**), (**F**), (**H**), and (**I**) are from three independent experiments; and in (**C**), (**D**), and (**G**) are from one experiment. Experiment replicates are shown in Figure S15.

Although inflammatory cytokines have been shown to induce the proteasomal degradation of PPARγ in adipocytes 43, 44, whether this phenomenon also occurs in macrophages is unclear. Here, we found that PPARγ levels were retained within the 2-h LPS treatment, regardless of nigericin treatment, and were decreased in response to the 4-h LPS treatment (Figure 6A-B). Because PPARγ has been reported to be cleaved by caspase-1 in tumor-associated macrophages 45, we tested whether the decreased PPARγ level was owing to cleavage by caspase-1. The observed reductions in the level of PPARγ could not be rescued by treatment with either the pan-caspase inhibitor (Z-VAD) or the caspase 1 inhibitor (YVAD) (Figure 6C). Interestingly, however, treatment with both the autophagy inhibitor, CQ, and the proteasome inhibitor, MG132, restored the reduced levels of PPARγ (Figure 6D-E). Consistent with previous studies 38, 40, CQ did not affect caspase-1 activation, whereas MG132 decreased caspase-1 activation in a dose-dependent manner (Figure S3B-C). These results suggest that PPARγ downregulation during NLRP3 inflammasome activation is likely mediated by autophagy or proteasomal degradation. Interestingly however, Signal-2 exposure treatment of rosiglitazone rarely restored the reduced PPARγ level (Figure 6F). These results raised the possibility of the off-target effect of rosiglitazone on attenuation of NLRP3 inflammasome activation.

### Rosiglitazone attenuated NLRP3 inflammasome activation in a PPARγ-independent mechanism

Because PPARγ is downregulated following LPS priming, this raised the question of how rosiglitazone acts via PPARγ to inhibit NLRP3*.* First, we performed rosiglitazone treatment following Signal-2 exposure protocol in peritoneal macrophages from* Pparg^C/-^* mice, which express approximately 30% of normal PPARγ levels in peritoneal macrophages owing to the presence of an unstable c-fos, AU-rich element in its 3′-UTR 32 (Figure S4 and S5A). Rosiglitazone, as well as pioglitazone, still effectively attenuated IL-1β and caspase-1 activation in *Pparg^C/-^* peritoneal macrophages (Figure 6G). This indicated that attenuation of the NLRP3 inflammasome by rosiglitazone can be achieved under a PPARγ hypomorphic condition. Second, we applied GW9662, an irreversible PPARγ antagonist that occupies the ligand binding site on Cys285 to prevent PPARγ activation by other PPARγ-binding ligands 46, in HEK293T cells. GW9662 co-treatment did not reverse the attenuation of IL-1β caused by either PPARγ overexpression or rosiglitazone treatment (Figure 6H and S5B). These results suggest that the inhibitory effect of rosiglitazone on the NLRP3 inflammasome is not dependent on ligand-mediated PPARγ activation. Finally, we applied a ligand binding site mutation of PPARγ at Pro467 to leucine (P467L), which is well documented for loss of ligand-mediated transactivation and inhibition of wild-type PPARγ in a dominant-negative manner 47, 48, in HEK293T cells. Co-transfection with the PPARγ P467L mutant as well as co-treatment of rosiglitazone with the PPARγ P467L mutant, retained the ability to attenuate NLRP3-dependent IL-1β production (Figure 6I and S5C), suggesting that attenuation of IL-1β production by rosiglitazone is not dependent on the ligand-binding pocket of PPARγ. In summary, these results suggest that rosiglitazone may potentially be working through a PPARγ-independent mechanism in attenuation of the NLRP3 inflammasome.

### PPARγ is required to limit NLRP3 inflammasome activation *in vivo*

**Figure 7:**
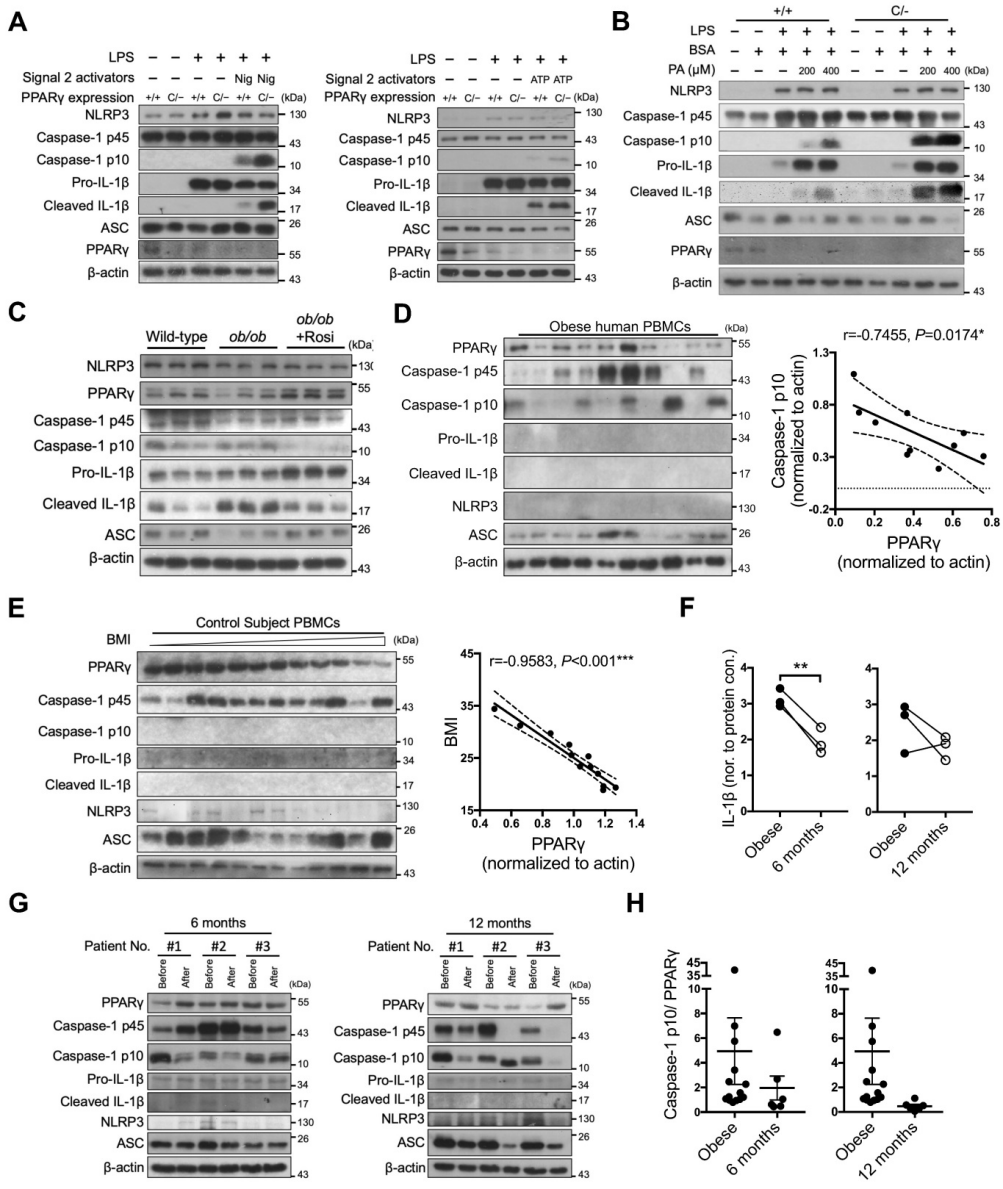
** PPARγ is required to limit NLRP3 inflammasome activation in mice and humans.** (**A-B**) Immunoblot analysis of caspase-1 activation and IL-1β maturation in LPS-primed wild-type (*Pparg^+/+^*) and *Pparg^C/-^* mouse peritoneal macrophages treated with (**A**) nigericin (Nig) and ATP, or with (**B**) palmitic acid (PA). (**C**) Immunoblot analysis of caspase-1 activation and mature IL-1β in the supernatant and NLRP3 inflammasome components and PPARγ in the cell lysates of lean control and *ob/ob* obese mouse peritoneal macrophages treated *ex vivo* with LPS and nigericin, as well as rosiglitazone, by Signal-2 exposure protocol. Each lane represents one independent experiment, and three independent experiments were included in this blot. (**D-E**) Immunoblot analysis of PPARγ, caspase-1 activation, mature IL-1β, NLRP3, and ASC in the cell lysates of peripheral blood mononuclear cells (PBMCs) from (**D**) obese and (**E**) control subjects. The correlation between caspase-1 activation and PPARγ level in the cell lysates of PBMCs from obese subjects. Spearman's rank correlation coefficients r and P value are provided. (**F**) IL-1β levels detected by ELISA in the cell lysates of PBMCs from obese subjects before and 6 (left panel) or 12 (right panel) months after weight-loss surgery. (**G**) Immunoblot analysis of PPARγ, caspase-1 activation, mature IL-1β, NLRP3, and ASC in the cell lysates of PBMCs from obese subjects before and 6 (left panel) or 12 (right panel) months after weight-loss surgery. (**H**) The ratio of caspase-1 activation to PPARγ in the cell lysates of PBMCs from obese subjects before and 6 (left panel) or 12 (right panel) months after weight-loss surgery. *P < 0.05 by paired *t*-test in (**F**). Representative blots in (**A**) and (**B**) are from three independent experiments. Experiment replicates are shown in Figure S16.

To investigate the *in vivo* physiological role of PPARγ in NLRP3 inflammasome activation, we used PPARγ hypomorphic* Pparg^C/-^* mice and evaluated the results obtained from immunoblotting and ELISA. NLRP3-dependent caspase-1 activation and IL-1β maturation were both increased in LPS-primed *Pparg^C/-^* macrophages stimulated with either nigericin or ATP (Figure 7A and S6A). Palmitic acid, a saturated fatty acid acting as a metabolic DAMP, has been reported to serve as a second signal for NLRP3 inflammasome activation 35. Accordingly, palmitic acid-induced caspase-1 activation and IL-1β maturation were increased in LPS-primed *Pparg^C/-^* macrophages (Figure 7B and S6B). These results suggest that PPARγ is involved in the regulation of metabolic DAMP-mediated NLRP3 inflammasome activation. Because nutrient overload is a major cause of increased levels of metabolic DAMPs and low-grade inflammation in obesity 49, we hypothesized that PPARγ could be a key factor in attenuating nutrient overload-induced inflammation. To determine how PPARγ affects the second signal of NLRP3 inflammasome activation, we isolated peritoneal macrophages from hyperphagic *ob/ob* mice (mimicking nutrient overload), and treated them with LPS and nigericin, as well as rosiglitazone, using the Signal-2 exposure protocol. Following LPS and nigericin treatment, we observed increased caspase-1 activation and IL-1β maturation, as evidenced by the immunoblotting and ELISA results, concomitant with a lower PPARγ level, in *ob/ob* peritoneal macrophages (Figure 7C and S6C). Moreover, the PPARγ level was restored, and both caspase-1 activation and IL-1β maturation were reduced in *ob/ob* macrophages treated *ex vivo* with rosiglitazone by Signal-2 exposure treatment (Figure 7C and S6C). These results suggest that PPARγ physiologically antagonizes NLRP3 inflammasome activation, especially under conditions of nutrient overload.

### Clinical relevance of PPARγ and NLRP3 inflammasome activation in obese patients

Finally, we investigated the correlation between PPARγ and NLRP3 inflammasome activation in obese patients by collecting peripheral blood mononuclear cells (PBMCs) before and 6 or 12 months after weight-loss surgery. We first examined PPARγ levels and NLRP3 inflammasome activation in PBMCs of obese patients before surgery. Although NLRP3 and IL-1β were barely detectable, we observed an inverse correlation between caspase-1 activation and PPARγ levels (*r* = -0.7109 and *P* < 0.05; Figure 7D). In addition, we included 12 control subjects with a BMI lower than 35 and without a diagnosis of metabolic syndrome, and found that the BMI and homeostasis model assessment-insulin resistance (HOMA-IR) of control subjects were significantly lower than those of obese patients (Figure S7A). Because mature caspase-1 and IL-1β were barely detectable in PBMCs of control subjects, we could not dissect the relationship between caspase-1 activation and PPARγ levels. However, we observed a negative correlation between BMI and PPARγ level (*r* = -0.9583, *P* < 0.001; Figure 7E) in control subjects. We subsequently compared the pre- and post-weight-loss-surgery levels of PPARγ and caspase-1 activation in PBMCs from the same obese patients. Following weight-loss surgery, the BMI and HOMA index of the obese patients were significantly decreased (Figure S7B). IL-1β levels were significantly decreased in the 6-month group (*P* = 0.0072) and tended to decrease in the 12-months group (*P* = 0.3131) (Figure 7F). Four of these patients (patients #1 and #2 from the 6-month group, and patients #1 and #3 from the 12-month group) displayed reduced caspase-1 activation and increased PPARγ levels after surgery when compared with those before surgery (Figure 7G). For patient #3 in the 6-month group and patient #2 in the 12-month group who did not present reduced caspase-1 activation, PPARγ levels after surgery did not increase either. Thus, weight-loss surgery tended to decrease the activated caspase-1 to PPARγ ratio in PBMCs of obese patients in both the 6-month and 12-month groups (*P* = 0.4903 and 0.3006, respectively) (Figure 7H).

## Discussion

Dysregulation of the NLRP3 inflammasome has been associated with various metabolic diseases such as obesity and type 2 diabetes 50, 51. However, how metabolic DAMP-associated NLRP3 inflammasome activation is attenuated remains unclear. Here, we found that PPARγ functions as an endogenous modulator that attenuates NLRP3 inflammasome activation in macrophages through interaction with NLRP3. The effects of PPARγ-mediated transrepression on NF-κB activity and IL-1β production are well documented 20; however, our study demonstrated that PPARγ also interferes with NLRP3 inflammasome formation by decreasing both NLRP3-ASC and NLRP3-NLRP3 interactions as well as NLRP3-dependent ASC oligomerization, and this interference is mediated *via* direct interaction between the DBD of PPARγ and the NBD and LRR of NLRP3. We further showed that PPARγ is required to limit metabolic DAMP-induced NLRP3 inflammasome activation in mouse macrophages. Finally, we demonstrated that a negative correlation exists between PPARγ and NLRP3 inflammasome activation in circulating mononuclear cells of obese patients.

PPARγ agonists are known to suppress LPS-induced inflammatory gene expression. Consistent with this, our results showed that the strongest effect on reducing IL-1β expression was observed when rosiglitazone was added before and throughout LPS/nigericin stimulation, suggesting that a transcriptional mechanism cannot be ruled out. Therefore, we modified the rosiglitazone treatment protocol to two stages, which have been used previously to dissect the effect of a variant of chemicals on inflammasome activation 52, 53. Although our results, as shown in Figure 1C, indicated that the expression of NLRP3 and pro-IL-1β is minimally affected in the Signal-2 exposure of rosiglitazone, it could not be assumed that Signal-1 stops acting as soon as LPS is washed out, and thus, that the rosiglitazone in the Signal-2 exposure could not affect Signal-1. In our study, it appears difficult to provide conclusive evidence that PPARγ directly regulates NLRP3 using *Pparg^C/-^* peritoneal macrophages or mice because it is difficult to rule out the transrepression effect of PPARγ on attenuation of Signal-1. Therefore, to minimize the effect of rosiglitazone and PPARγ on the expression of NLRP3 and pro-IL-1β, we applied a HEK293T system with reconstitution of each inflammasome component. This system expresses NLRP3 and pro-IL-1β, which are driven by CMV promoters, to minimize the influence of NF-κB-dependent transcription. Consistent with this, we also found that expression of PPARγ down-regulates the secretion of cleaved IL-1β in this reconstituted HEK293T system. Thus, these two systems unequivocally suggest a direct inhibitory role of PPARγ in the activation of the NLRP3 inflammasome. It is worth noting that genetic loss of PPARγ did not elicit higher IL-1β induction by LPS (Figure 7A-B). The relatively similar levels of LPS-induced NLRP3 and pro-IL-1β between *Pparg^C/-^* and *Pparg^+/+^* peritoneal macrophages could be related to a short-term treatment protocol of LPS (3.5 h).

Moreover, we found that the distribution of nuclear receptor PPARγ is altered during NLRP3 inflammasome activation. PPARγ was mainly distributed in both the nucleus and cytosol (63% cytosolic PPARγ expression) in non-treated control macrophages, whereas LPS and nigericin treatment induced a predominantly cytosolic PPARγ expression (nearly 80%) (Figure S8). These results suggest that the cellular distribution of PPARγ is altered during NLRP3 inflammasome activation, implicating a role of PPARγ in the cytosol where the NLRP3 inflammasome is located.

The LBD of PPARγ is known to interact with the LXXLL motif of its transcriptional coactivators, such as NCOA/SRC 54. Interestingly, we found that NLRP3 contains four LXXLL motifs, two of which are in the LRR and two in the NBD. Therefore, we initially hypothesized that the leucine-rich motif in the LRR of NLRP3 could be a candidate region mediating the interaction between NLRP3 and the LBD of PPARγ. However, this was not supported by the results of the co-immunoprecipitation of PPARγ with NLRP3-truncated variants. Instead, we found that PPARγ interacts with NLRP3 through its DBD. Although the DBDs of nuclear receptors, which contain two zinc fingers, recognize DNA response elements 55, they are also reported to be involved in protein-protein interactions 56-60. Therefore, it is plausible to support a novel mechanism of PPARγ in the regulation of the NLRP3 inflammasome, which is mediated through a physical interaction with its DBD.

In this study, we found that both the NBD and LRR of NLRP3 were involved in mediating its interaction with PPARγ. Interestingly, other proteins, such as TXNIP and NEK7, also bind the NBD and LRR of NLRP3 to regulate NLRP3 inflammasome activity 15, 16, 61. This raises the question of why the NBD and LRR of NLRP3 are concurrently targeted by other proteins? NLRP3 NBD is a multifunctional domain involved in NLRP3 oligomerization and nucleotide binding 14, 62, implicating NBD as a target for regulating NLRP3 inflammasome activity. In addition to the NBD, the LRR of NLRP3 is also involved in its interaction with PPARγ. The LRR recognizes numerous stimuli and acts as a primary sensing domain in most NLR proteins 13. Several studies have demonstrated the importance of, and requirement for, the LRR in NLRP3 inflammasome activation, particularly through interaction with NLRP3 inflammasome-promoting factors 63, 64. Combined, these studies suggest that physical interaction with the NBD and LRR is critical for the assembly and activation of the NLRP3 inflammasome. Therefore, it is likely that PPARγ inhibits NLRP3 inflammasome activation, at least in part, through the steric hindrance of the binding of the NBD and LRR to NLRP3 inflammasome-promoting factors.

Our study demonstrated that PPARγ interacts primarily with NLRP3, but not ASC, suggesting that PPARγ does not affect other ASC-associated inflammasomes. Consistent with this observation, Signal-2 exposure treatment did not reduce AIM2 and NLRC4 inflammasome activation, suggesting that PPARγ specifically targets the NLRP3 inflammasome. Although NLRC4 also contains an NBD and an LRR, a common feature of the NLR protein family 65, NBD and LRR sequence analysis showed that there is less than 30% identity between NLRC4 and NLRP3 (Table S4). This result further supports the specificity of the interaction between PPARγ and the NLRP3 inflammasome. Moreover, Signal-2 exposure treatment also attenuated the production of IL-1β and activation of caspase-1, which was triggered not only by nigericin, but also by MSU, alum, and ATP. These findings implicate PPARγ as a general regulator that modulates NLRP3 inflammasome activation induced by various stimuli. Whether the interaction between PPARγ and NLRP3 exists in the untreated, basal stage is interesting. In untreated macrophages, the interaction between PPARγ and NLRP3 is difficult to address because untreated macrophages express very little NLRP3 (Figure 3F). Therefore, we performed reverse co-immunoprecipitation by precipitating PPARγ and detecting NLRP3. Our results showed that PPARγ co-immunoprecipitated with NLRP3 in untreated, control macrophages (Figure S2), suggesting that the interaction between NLRP3 and PPARγ exists in the untreated stage. Their colocalization in the basal stage was also confirmed by immunofluorescence staining and PLA of PPARγ and NLRP3.

While LPS has been shown to reduce PPARγ levels, it is linked to an NF-κB-dependent attenuation in mRNA synthesis 34. Consistent with this, we found that PPARγ protein levels were decreased in response to LPS treatment regardless of the second signal of NLRP3 inflammasome activation in both *Pparg^+/+^* and *Pparg^C/-^* mouse peritoneal macrophages. As NLRP3 inflammasome activation is an extremely rapid event leading to substantial pyroptosis within 30 min to 1 h 66, 67, we analyzed PPARγ protein levels within this timeframe. Our results showed that PPARγ levels were retained within the 2-h LPS treatment, regardless of nigericin treatment. However, PPARγ levels were decreased after the 4-h LPS treatment (Figure 6A). Because PPARγ levels are retained within the 2-h LPS treatment, it is possible that PPARγ can modulate the NLRP3 inflammasome within this timeframe. In adipocytes, inflammatory cytokines have been shown to induce caspase-mediated cleavage and proteasomal degradation of PPARγ 43, 44. However, whether the levels of PPARγ are regulated through caspase-mediated cleavage and/or proteasomal degradation in macrophages remains unclear. In the current study, exposure to pan-caspase and caspase-1 inhibitors did not inhibit PPARγ degradation during inflammasome activation. Furthermore, PPARγ containing a D62A mutation and proposed to be resistant to caspase-1 cleavage 68, did not further attenuate the production of cleaved IL-1β in NLRP3 inflammasome reconstituted cells (Figure S1C). This indicates that downregulation of PPARγ is not associated with caspase-1-mediated degradation during inflammasome activation. In contrast, treatment with the proteasome inhibitor MG132 or the autophagy inhibitor CQ reversed PPARγ degradation, suggesting that PPARγ is degraded via either the proteasomal or autophagic pathway during inflammasome activation.

Combined, our results demonstrated that a regulatory feedback loop exists between PPARγ and inflammasome activation in macrophages. Within this loop, PPARγ represses NLRP3-mediated inflamemasome activation to properly regulate the inflammatory status and allow the execution of PPARγ function. In response to inflammatory stimuli, downregulation of PPARγ levels via either the transcriptional attenuation 34 or proteasomal/autophagic pathway in our study limits its anti-inflammatory action, thus allowing further activation of the NLRP3 inflammasome. This shows that the interplay between PPARγ and NLRP3 is important to limit aberrant IL-1β-mediated inflammatory responses. Thus, PPARγ serves as an endogenous modulator for NLRP3 inflammasome activation, and removal of PPARγ is critical to complete activation of the NLRP3 inflammasome.

Pharmacologically, selective PPARγ agonists, including rosiglitazone and pioglitazone, have been shown to decrease NLRP3 inflammasome activation 30, 69. Although ligand-dependent SUMOylation of PPARγ helps explain the attenuation of NF-κB activity in the nucleus 21, how PPARγ agonists reduce NLRP3 inflammasome activation remains unclear. While rosiglitazone enhances both the transcriptional activity and expression levels of PPARγ 70, we found that Signal-2 exposure treatment with rosiglitazone as well as another TZD drug pioglitazone, rarely restored the levels of PPARγ (Figure 6F-G). To dissect the working timeframe of rosiglitazone, we performed treatments with rosiglitazone following the Signal-2 exposure protocol with several timeframes (0.5, 1, and 2 h) in mouse peritoneal macrophages. Our results showed that the inhibitory effect of rosiglitazone took place in the 2-h treatment group, although PPARγ levels remained quite low at this time point (Figure S5D). These results suggest that the inhibitory effect of rosiglitazone on the NLRP3 inflammasome may be working through a PPARγ-independent mechanism. In addition to the TZD drugs, 15-deoxy-Δ12,14-prostaglandin J2 (15d-PGJ2), an endogenous PPARγ agonist with distinct chemistry from that of TZD drugs, also effectively down-regulated IL-1β and caspase-1 activation in both *Pparg^+/+^* and *Pparg^C/-^* peritoneal macrophages (Figure S5E). Interestingly, 15d-PGJ2 modestly restored PPARγ levels in both *Pparg^+/+^* and *Pparg^C/-^* peritoneal macrophages. Nevertheless, this highlights PPARγ agonism as a therapeutic option for targeting NLRP3 inflammasome activation in NLRP3-related metabolic diseases.

Th2 cytokines, such as IL-4, have been shown to suppress inflammasome activation and increase PPARγ levels 71. However, our results showed that IL-4 treatment following Signal-2 exposure did not affect IL-1β maturation and caspase-1 activation, and did not reverse PPARγ expression in this short-term (6.5 h) treatment (Figure S9A). The treatment protocol followed by Huang *et al*. showed that long-term (48 h) treatment of IL-4 increased PPARγ levels in macrophages 71; therefore, we further performed long-term treatment with IL-4 following the Signal-2 exposure protocol. Our results showed that treatment with IL-4 for 48 and 72 h decreased IL-1β and caspase-1 activation and modestly reversed PPARγ levels, particularly in the 72-h treatment group (Figure S9B). Thus, although long-term treatment with IL-4 suppressed inflammasome activation and modestly increased PPARγ protein levels, short-term treatment with IL-4 did not affect inflammasome activation and PPARγ levels.

We observed a negative correlation between PPARγ and NLRP3 inflammasome activation in both mouse and human macrophages. Interestingly, in PPARγ-defective macrophages (*Pparg^C/-^*), NLRP3 inflammasome activation induced by nigericin, ATP, or PA was increased compared to that in WT macrophages (*Pparg^+/+^*). Moreover, increased NLRP3 inflammasome activation and decreased PPARγ levels were found in LPS + nigericin-treated macrophages from *ob/ob* mice (Figure 7C and S6C). To clarify whether the downregulation of PPARγ in *ob/ob* macrophages observed in Figure 7C resulted from obesity itself or LPS + nigericin treatment, we examined basal, untreated PPARγ levels from control and *ob/ob* macrophages, and found that basal, untreated PPARγ levels remained similar between the two groups (Figure S6D-E). These results suggest that the downregulation of PPARγ in *ob/ob* macrophages does not result from obesity itself. In addition, we found reduced levels of NLRP3 and ASC in the lysate from LPS + nigericin-treated *ob/ob* macrophages (Figure 7C). We further found that the supernatant from *ob/ob* macrophages exhibited significantly higher ASC levels and tended to have higher NLRP3 levels than wild-type macrophages (Figure S6F-G), suggesting that these proteins were released from *ob/ob* macrophages upon NLRP3 inflammasome activation.

Although IL-1β was barely detectable in unstimulated PBMCs obtained from obese patients, we found a negative correlation between the level of PPARγ and caspase-1 activation. To exclude confounding factors resulting from individual variation, we examined these parameters in the same subjects both before and after weight-loss surgery. Indeed, we observed unchanged PPARγ levels after surgery in some patients, and our results concurrently showed no change in caspase-1 activation in these patients (Figure 7G). Instead, patients with increased PPARγ displayed reduced caspase-1 activation. These results support a negative correlation between PPARγ and NLRP3 inflammasome activation. Importantly, the ratio of mature caspase-1 to the level of PPARγ markedly decreased after surgery. Therefore, the ratio of mature caspase-1 and the level of PPARγ can be represented as an “NLRP3-accelerating index” in obesity. These findings confirmed that PPARγ functions as an endogenous modulator of NLRP3 inflammasome activation. In the current study, it was difficult to separate the role of PPARγ in the non-transcriptional regulation of the NLRP3 inflammasome. Indeed, owing to the nature of human study, we applied correlation analysis for the study between the PPARγ and NLRP3 inflammasomes. Moreover, rosiglitazone/pioglitazone is often used as a second-line therapy for patients with type 2 diabetes 72. Because the confounding factors in patients who take rosiglitazone/pioglitazone tend to be more, it is difficult to select control subjects and to interpret the cause owing to the use of other anti-diabetic drugs. Thus, the causal relationship of this correlation remains elusive. Whether this correlation is caused by a reduction in inflammatory signals or a change in PPARγ expression requires further study.

Although several compounds have shown potential to inhibit NLRP3 inflammasome activation, their clinical availability remains limited 1, 73-76. Our study demonstrated an additional anti-inflammatory role for PPARγ that specifically targets NLRP3 inflammasome activation. PPARγ serves as an endogenous modulator for NLRP3 inflammasome activation by interacting with NLRP3 and interfering with inflammasome complex assembly. The PPARγ agonist, currently clinically available, efficiently attenuates NLRP3 inflammasome activation. Our study not only identified a novel therapeutic application for PPARγ in addition to its canonical role as a transrepressor of NF-κB activity, but may also facilitate the pharmacological development of therapeutic agents targeting NLRP3 inflammasome-related diseases.

## Supplementary Material

Supplementary figures and tables.

## Abbreviations

ASC: apoptosis-associated speck-like protein containing a caspase activation and recruitment domain
AF-1: activating function-1
AF-2: activating function-2
ATP: adenosine triphosphate
AIM2: absent in melanoma 2
CGI-58: comparative gene identification 58
DAMPs: damage-associated molecular patterns
DBD: DNA-binding domain
dsDNA: double stranded DNA
He3: Helix 3
LBD: ligand-binding domain
LRR: leucine-rich-repeat domain
MSU: monosodium urate
NBD: nucleotide-binding domain
NLRP3: nucleotide-binding oligomerization domain-like receptors (NLRs) pyrin domain containing 3
NLRC4: NLR family CARD domain containing 4
NEK7: NIMA related kinase 7
OE: overexpression
PPARγ: peroxisome proliferator activated receptor gamma
PBMCs: peripheral blood mononuclear cells
PYD: pyrin domain
PLA: proximity ligation assay
ROS: reactive oxygen species
Rosi: Rosiglitazone
SN: supernatant
TXNIP: thioredoxin interacting protein
WT: wild-type
WCL: whole cell lysate
