# Supplementary material for: Inhibitory effect of PPARγ on NLRP3 inflammasome activation
Source: Theranostics. 2021 Jan 1;11(5):2424–41. doi: 10.7150/thno.46873 (PMC7797672; doi:10.7150/thno.46873)
Supplement: Supplementary file 1 — Supplementary figures and tables. [file thnov11p2424s1.pdf]

Supplementary Figures

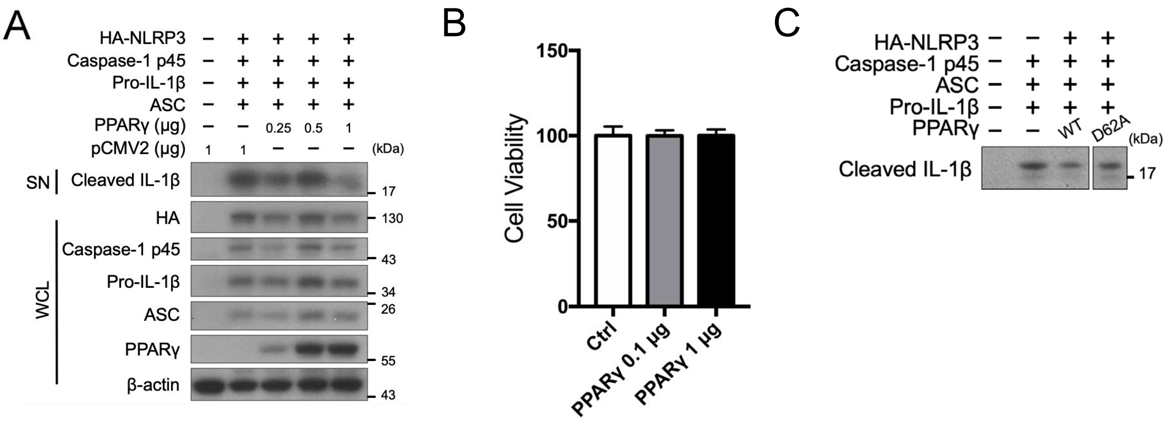

Figure S1. PPARγ expression attenuated the production of cleaved IL-1β in NLRP3 inflammasome reconstituted HEK293T cells.

(**A**) Immunoblot analysis of mature IL-1β in the supernatant (SN) and indicated components and PPARγ in whole cell lysates (WCL) of NLRP3 inflammasome-reconstituted HEK293T cells transfected with indicated components. IL-1β production was significantly decreased in 1 μg PPARγ transfection group. (**B**) MTT assay of HEK293T cells transfected with control (Ctrl, pCMV2), 0.1 μg or 1 μg PPARγ expression plasmids (n = 3 in each group). Transfection of 0.1 and 1 μg PPARγ did not change the cell viability. (**C**) Immunoblot analysis of mature IL-1β in the supernatant of NLRP3 inflammasome-reconstituted HEK293T cells expressing wild-type and D62A mutant PPARγ.

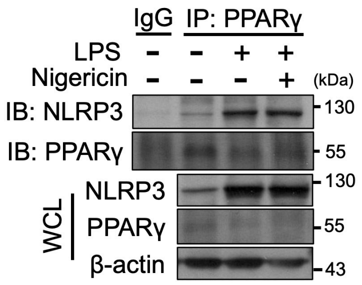

Figure S2. PPARγ interacted with NLRP3 in mouse peritoneal macrophages. Immunoprecipitation and immunoblot analysis of the interaction between NLRP3 and PPARγ in mouse peritoneal macrophages. Reverse co-immunoprecipitation was performed by precip-itating PPARγ and detecting NLRP3.

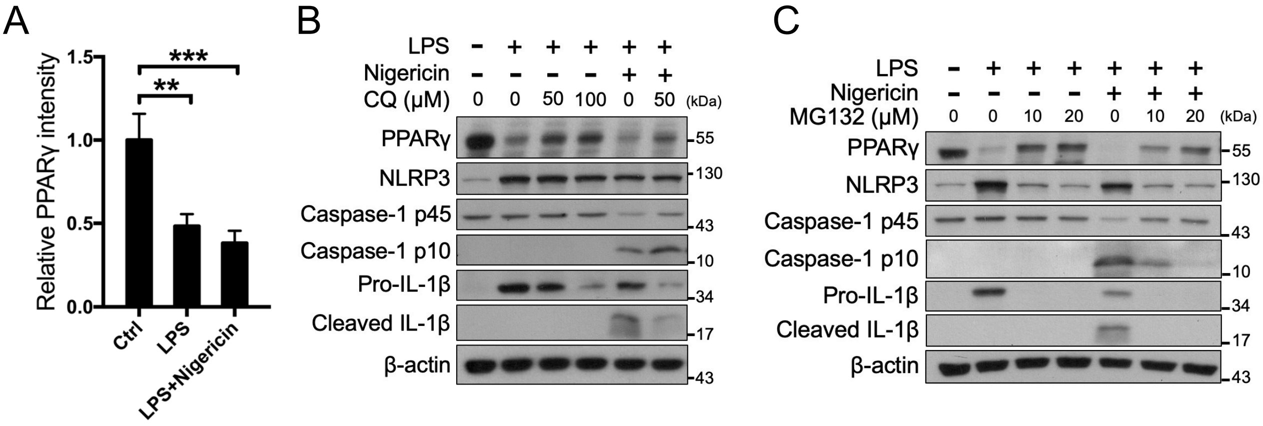

Figure S3. MG132, but not CQ, attenuated NLRP3 inflammasome activation in mouse peritoneal macrophages.

(**A**) Quantification of PPARγ (*green*) intensity per cell in mouse peritoneal macrophages from Figure 3G (n = 6 in each group). (**B** and **C**) Immunoblot analysis of caspase-1 activation and IL-1β maturation in mouse peritoneal macrophages treated with (**B**) chloroquine (CQ) and (**C**) MG132 with indicated concentration. $^{**}P < 0.01$ and$^{***}P < 0.001$ by one-way ANOVA with Fisher's LSD test.

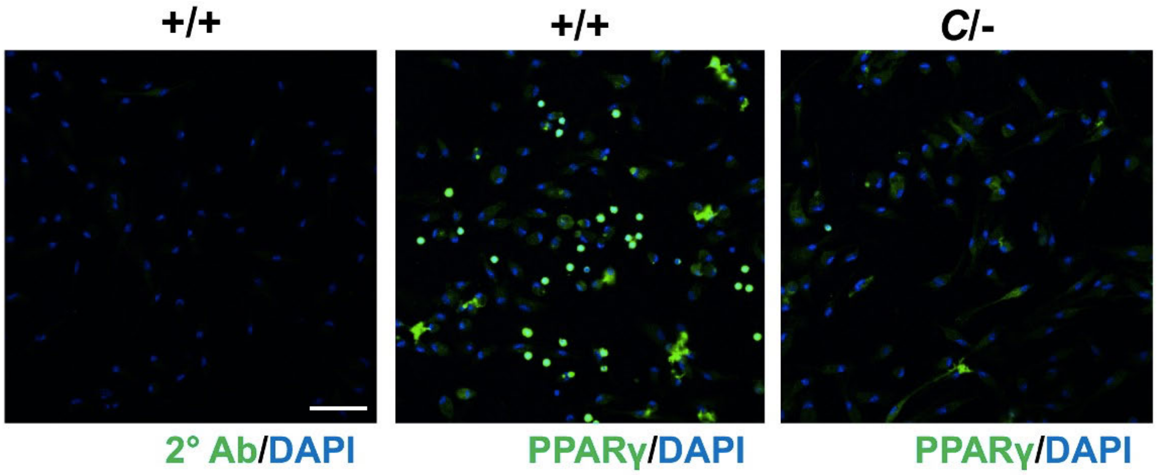

Figure S4. PPARγ expression was decreased in *Pparg*$^{C/-}$ mouse peritoneal macrophages. Immunofluorescent staining of PPARγ (*green*) in *Pparg*$^{+/+}$ and *Pparg*$^{C/-}$ mouse peritoneal macrophages. The control is no primary antibody followed by incubation with secondary antibodies and detection reagents. Scale bar, 50 μm.

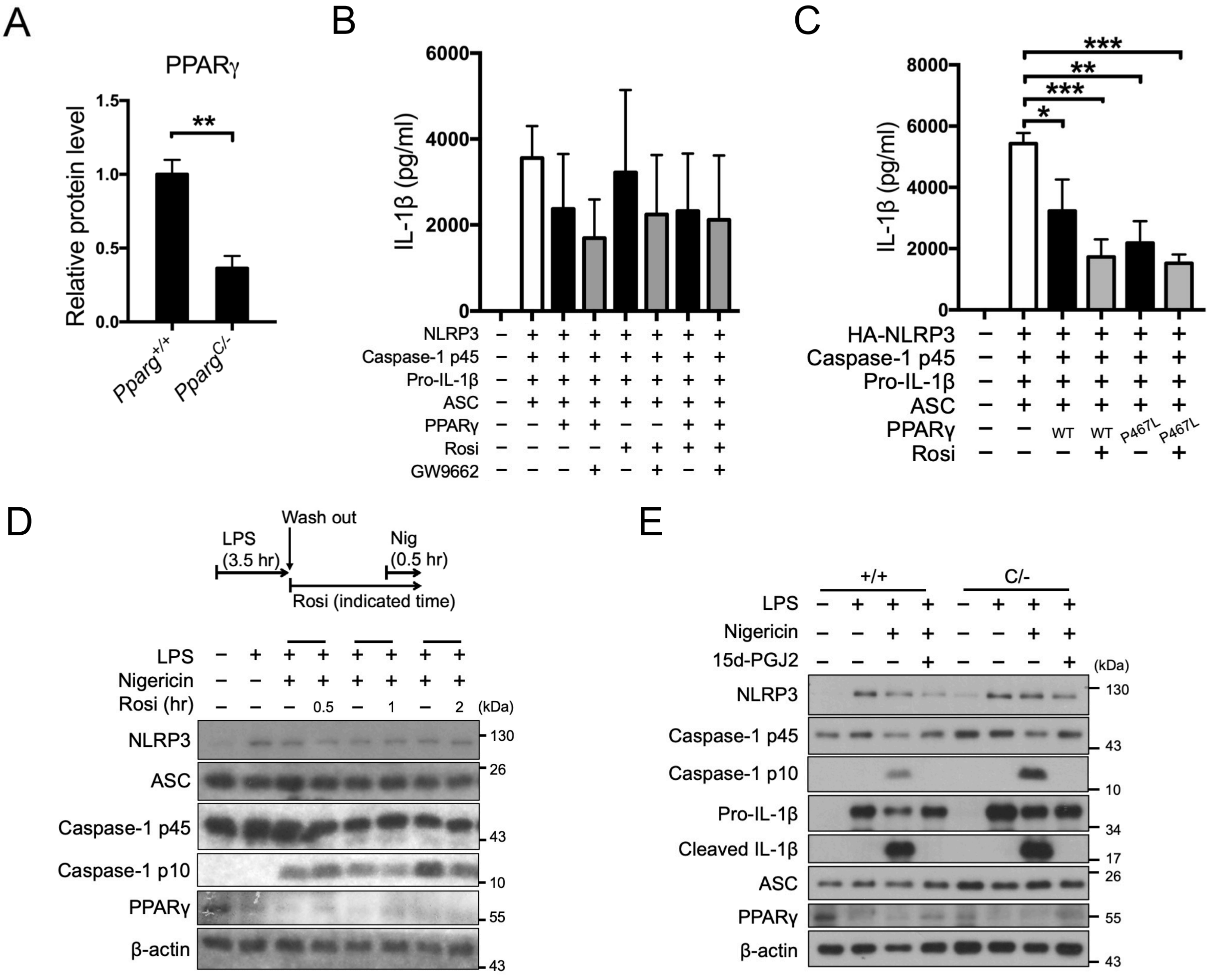

Figure S5. Rosiglitazone attenuated NLRP3 inflammasome through a PPARγ-independent mechanism.

(**A**) Quantification of PPARγ protein level in untreated, control wild-type (*Pparg*$^{+/+}$) and *Pparg*$^{C/-}$ mouse peritoneal macrophages from Figure 6G, 7A, and 7B. (B and **C**) IL-1β levels in the culture medium were detected by ELISA related to Figure 6H and 6I. Culture medium was collected from NLRP3 inflammasome-reconstituted HEK293T cells transfected with indicated components and PPARγ (WT and P467L mutant). Rosiglitazone (Rosi, 20 μM) and GW9662 (20 μM) were treated for 24 h after transfection. Three independent experiments were included. (**D**) Immunoblot analysis of caspase-1 activation and IL-1β maturation in mouse peritoneal macrophages treated with rosiglitazone (Rosi, 20μM) for indicated time by Signal-2 exposure protocol. A schematic diagram on the top shows the experimental design of co-treatment with Rosi by Signal-2 exposure protocol. (E) Immunoblot analysis of caspase-1 activation and IL-1β maturation in *Pparg*$^{+/+}$ and *Pparg*$^{C/-}$ mouse peritoneal macrophages treated with 15d-PGJ2 (2.5 μM) by Signal-2 exposure protocol. **$P < 0.01$ by *t*-test in (**A**). *$P < 0.05$, **$P < 0.01$ and ***$P < 0.001$ by one-way ANOVA with Fisher's LSD test in (**C**).

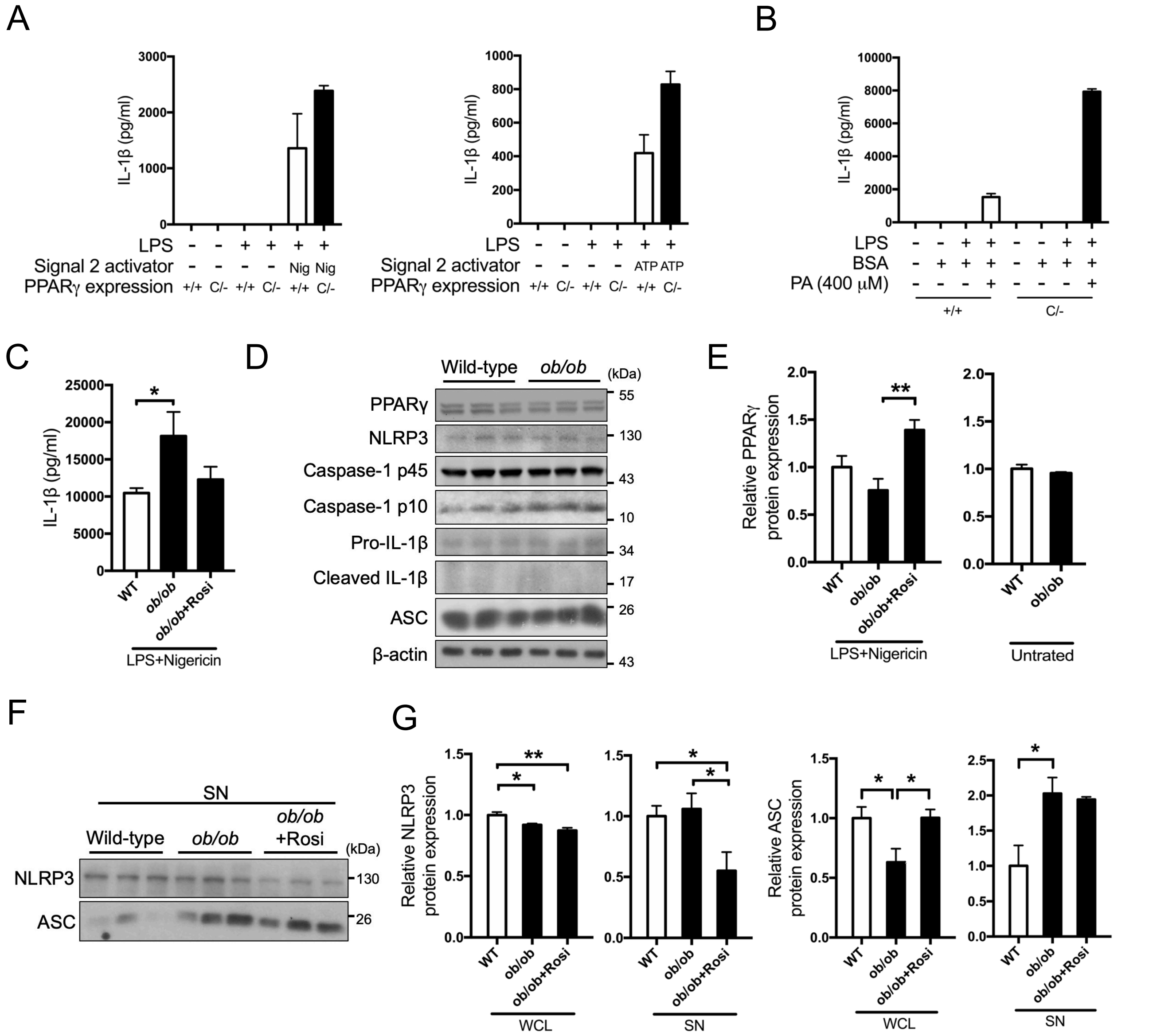

Figure S6. PPARγ is required to limit NLRP3 inflammasome activation in mice. (**A** and **B**) IL-1β levels in the culture medium were detected by ELISA from Figure 7A and 7B. Culture medium was collected from LPS-primed wild-type (*Pparg*$^{+/+}$) and *Pparg*$^{C/-}$ mouse peritoneal macrophages treated with (**A**) nigericin (Nig) and ATP, or with (**B**) palmitic acid (PA). Two independent experiments were included. (**C**) IL-1β levels in the culture medium were detected by ELISA from Figure 7C. Culture medium was collected from lean control and *ob/ob* obese mouse peritoneal macrophages treated *ex vivo* with LPS and nigericin, as well as rosiglitazone, by Signal-2 exposure protocol. Three independent experiments were included. (**D**) Immunoblot analysis of caspase-1 activation and mature IL-1β in the supernatant and NLRP3 inflammasome components and PPARγ in the cell lysates of untreated, basal lean control and *ob/ob* obese mouse peritoneal macrophages. The supernatant was collected after 10 h culture

in FBS-free RPMI medium. Three independent experiments were included in this blot. (**E**) Quantification of PPARγ protein levels in the cell lysates of LPS+nigericin treated (from Figure 7C) or untreated (from Figure S6D) lean control and *ob/ob* obese mouse peritoneal macrophages with Signal-2 exposure protocol. (**F**) Immunoblot analysis of NLRP3 and ASC in the supernatant of lean control and *ob/ob* obese mouse peritoneal macrophages treated *ex vivo* with LPS and nigericin, as well as rosiglitazone, by Signal-2 exposure protocol from Figure 7C. (**G**) Quantification of NLRP3 (left panels) and ASC (right panels) protein levels in the whole cell lysate (WCL) and supernatant (SN) of lean control and *ob/ob* obese mouse peritoneal macrophages treated *ex vivo* with LPS and nigericin, as well as rosiglitazone, by Signal-2 exposure protocol from Figure 7C and S6F. *$P < 0.05$ and **$P < 0.01$ by one-way ANOVA with Fisher's LSD test.

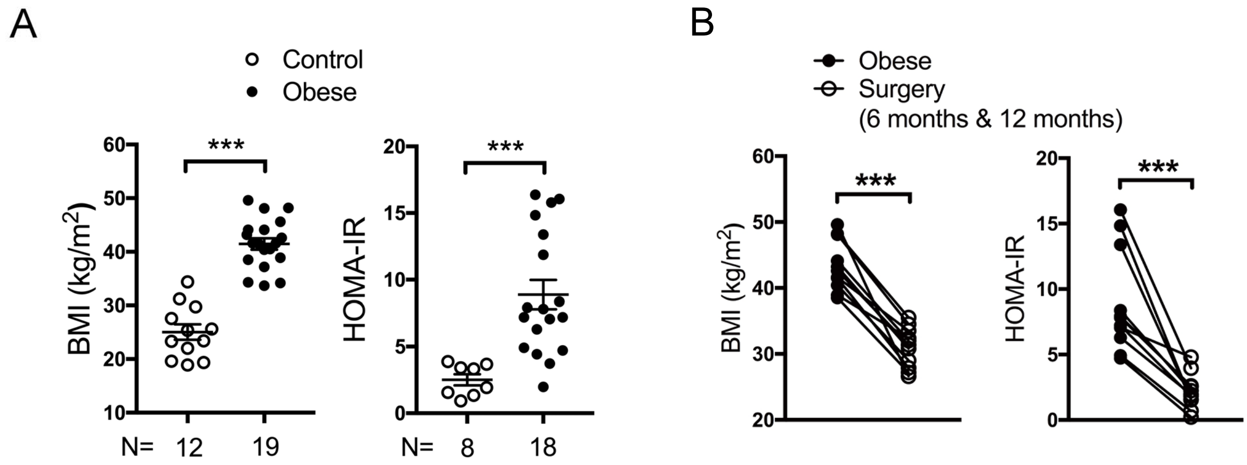

Figure S7. BMI and HOMA-IR were changed between obese patients and control subjects.

(**A**) BMI and HOMA-IR of control and obese subjects before weight-loss surgery. (**B**) BMI and HOMA-IR of obese subjects before (Obese) and 6 or 12 months after (Surgery) weight-loss surgery. ***P < 0.001 by t-test in (**A**), and ***P < 0.001 by paired t-test in (**B**).

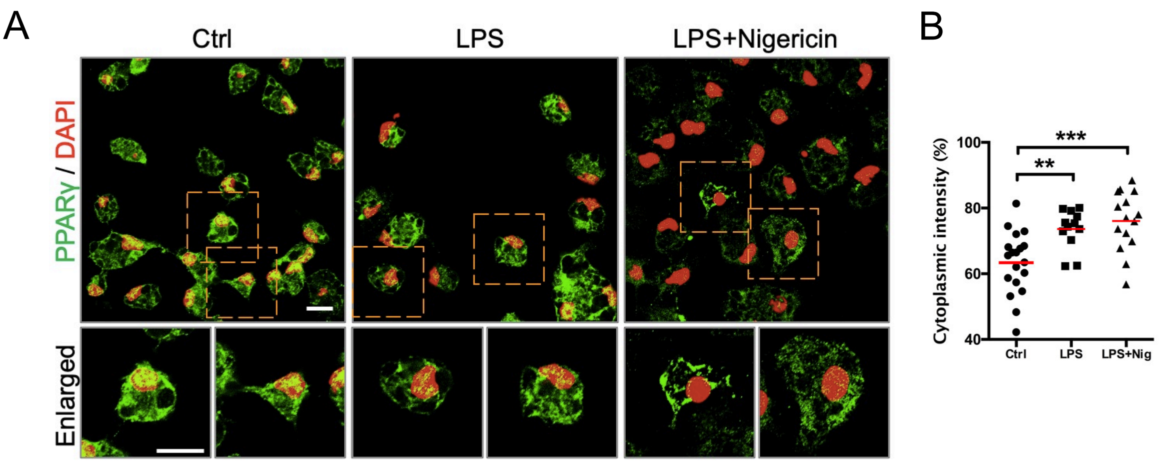

Figure S8. Cellular distribution of PPARγ is altered during NLRP3 inflammasome activation.

(**A**) Immunofluorescent staining of PPARγ (*green*) and DAPI (*red*) in mouse peritoneal macrophages of control, LPS, and LPS+nigericin groups. Scale bar, 10 µm. (**B**) Quantification of PPARγ intensity in the cytosol by the TissueFAXS fluorescence analysis module. $^{**}P < 0.01$ and $^{***}P < 0.001$ by one-way ANOVA with Fisher's LSD test.

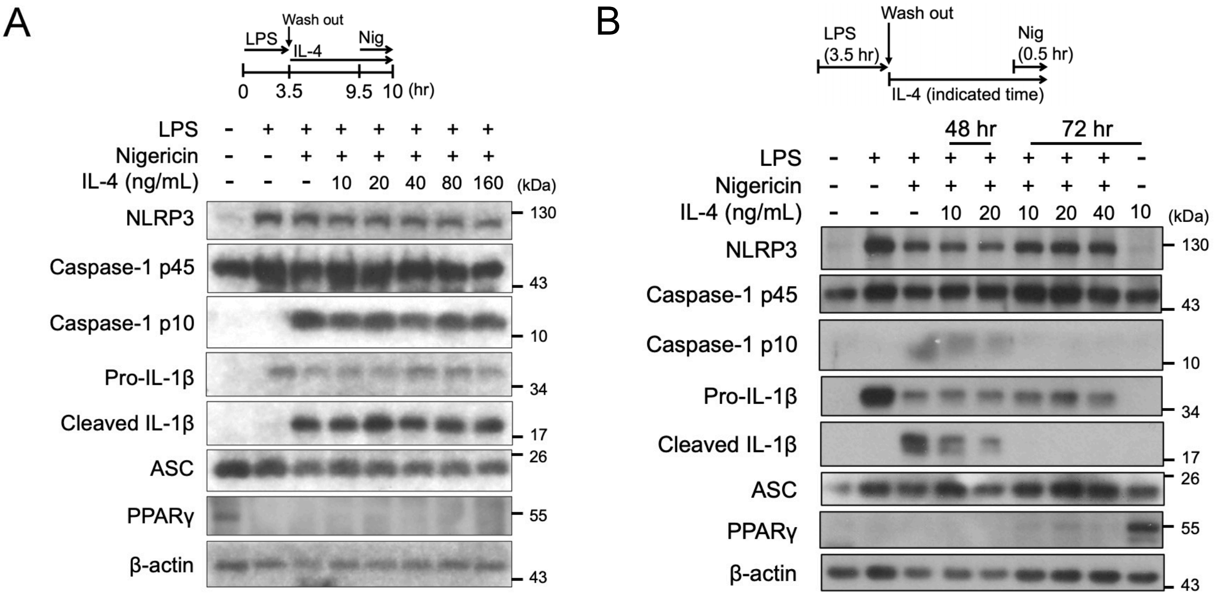

Figure S9. Long-term treatment, but not short-term treatment, of IL-4 suppressed NLRP3 inflammasome activation and modestly increased PPARγ protein level.

(**A** and **B**) Immunoblot analysis of caspase-1 activation and IL-1β maturation in mouse peritoneal macrophages treated with IL-4 for (**A**) 6.5 h and (**B**) 48 and 72 h by Signal-2 exposure protocol. Schematic diagrams on the top show the experimental design of co-treatment with IL-4 in the (**A**) short-term and (**B**) long-term protocol of NLRP3 inflammasome activation.

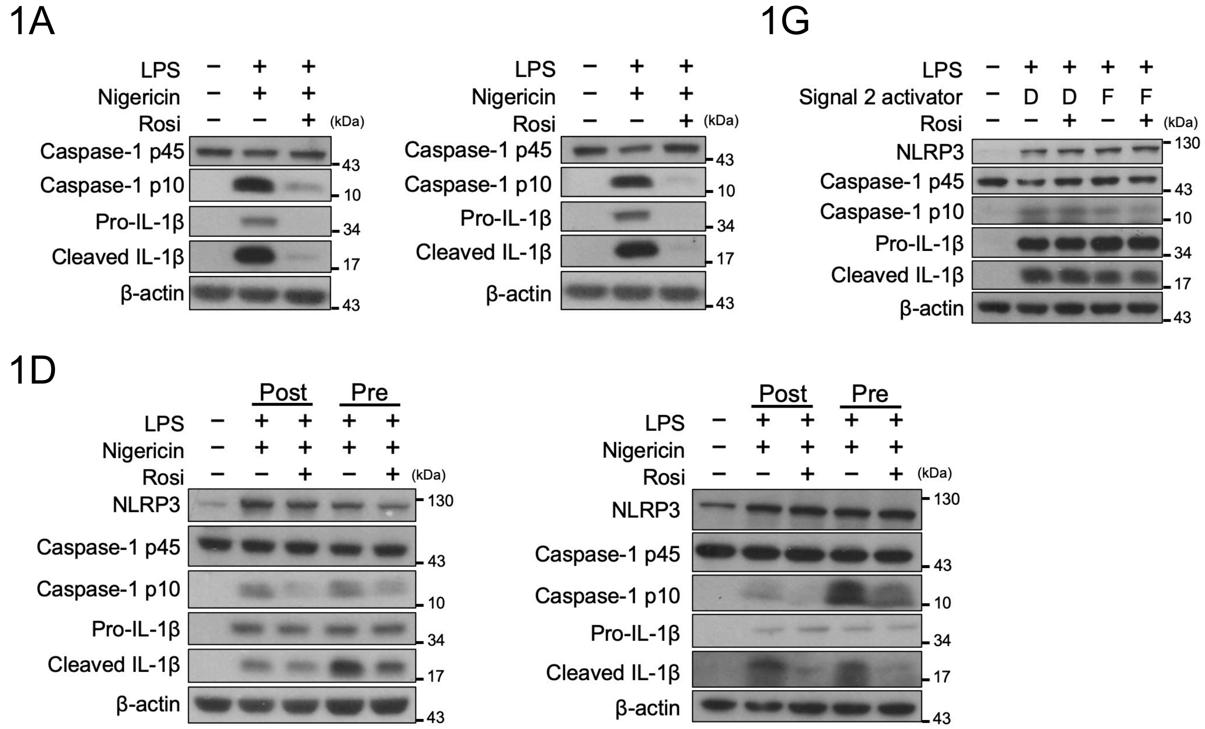

Figure S10. Repeated data of the immunoblotting related to Figure 1.

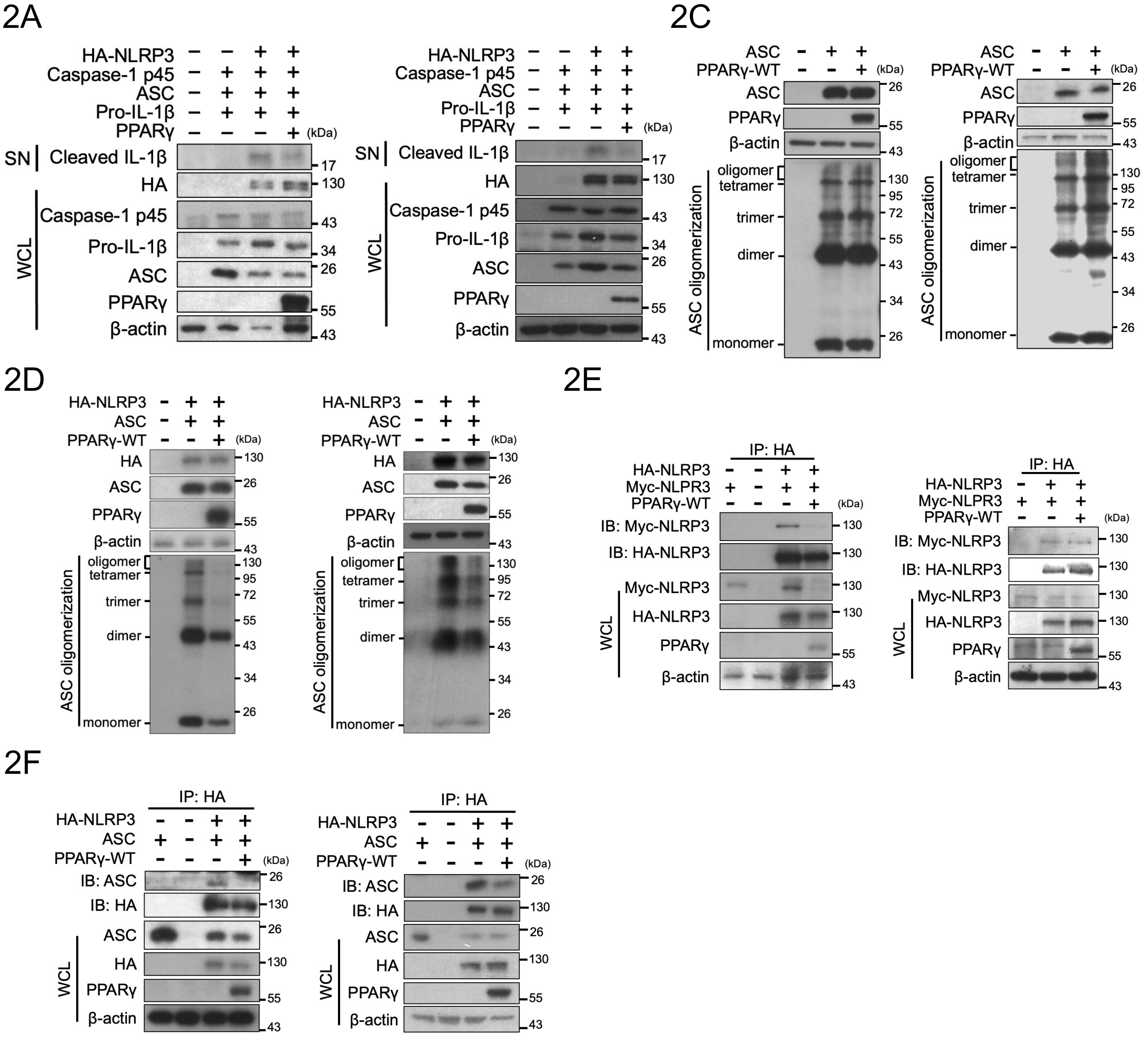

Figure S11. Repeated data of the immunoblotting related to Figure 2.

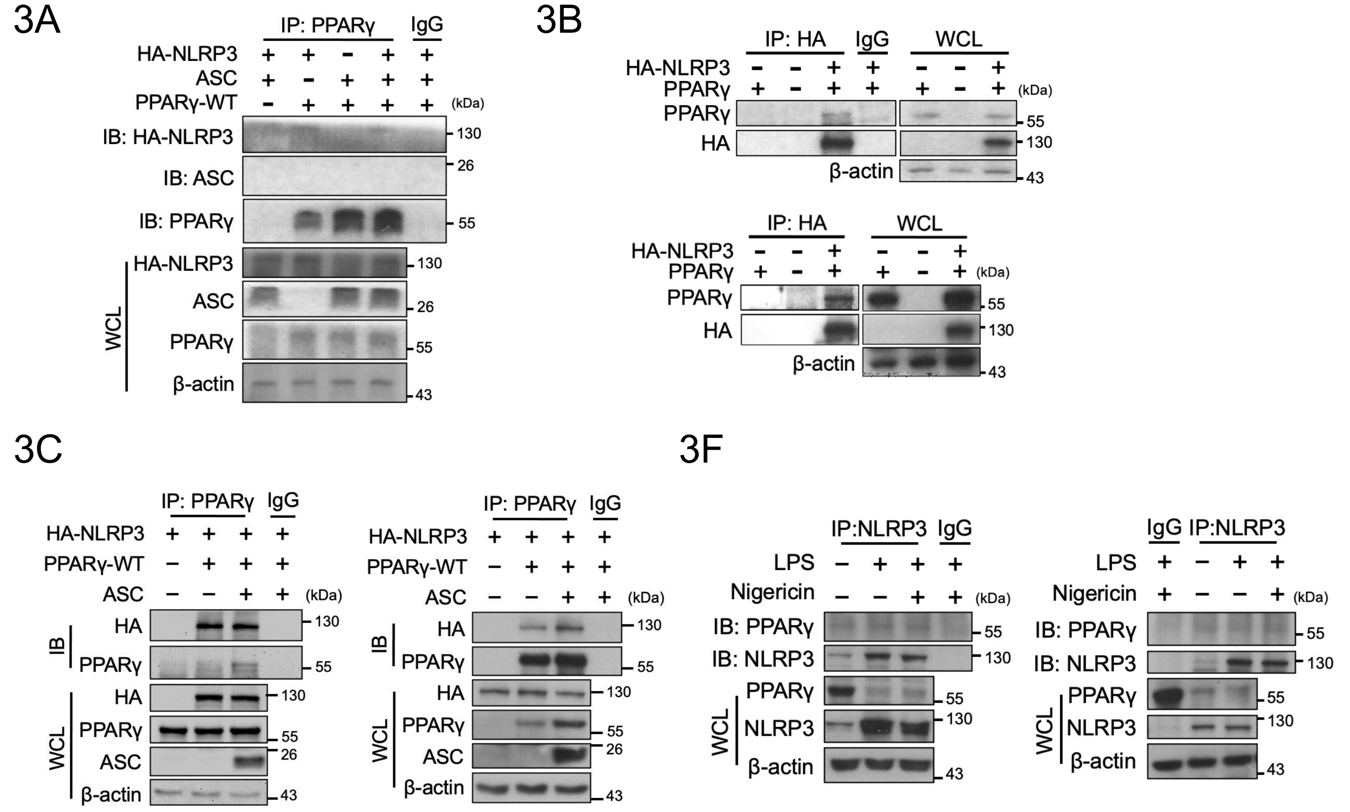

Figure S12. Repeated data of the immunoblotting related to Figure 3.

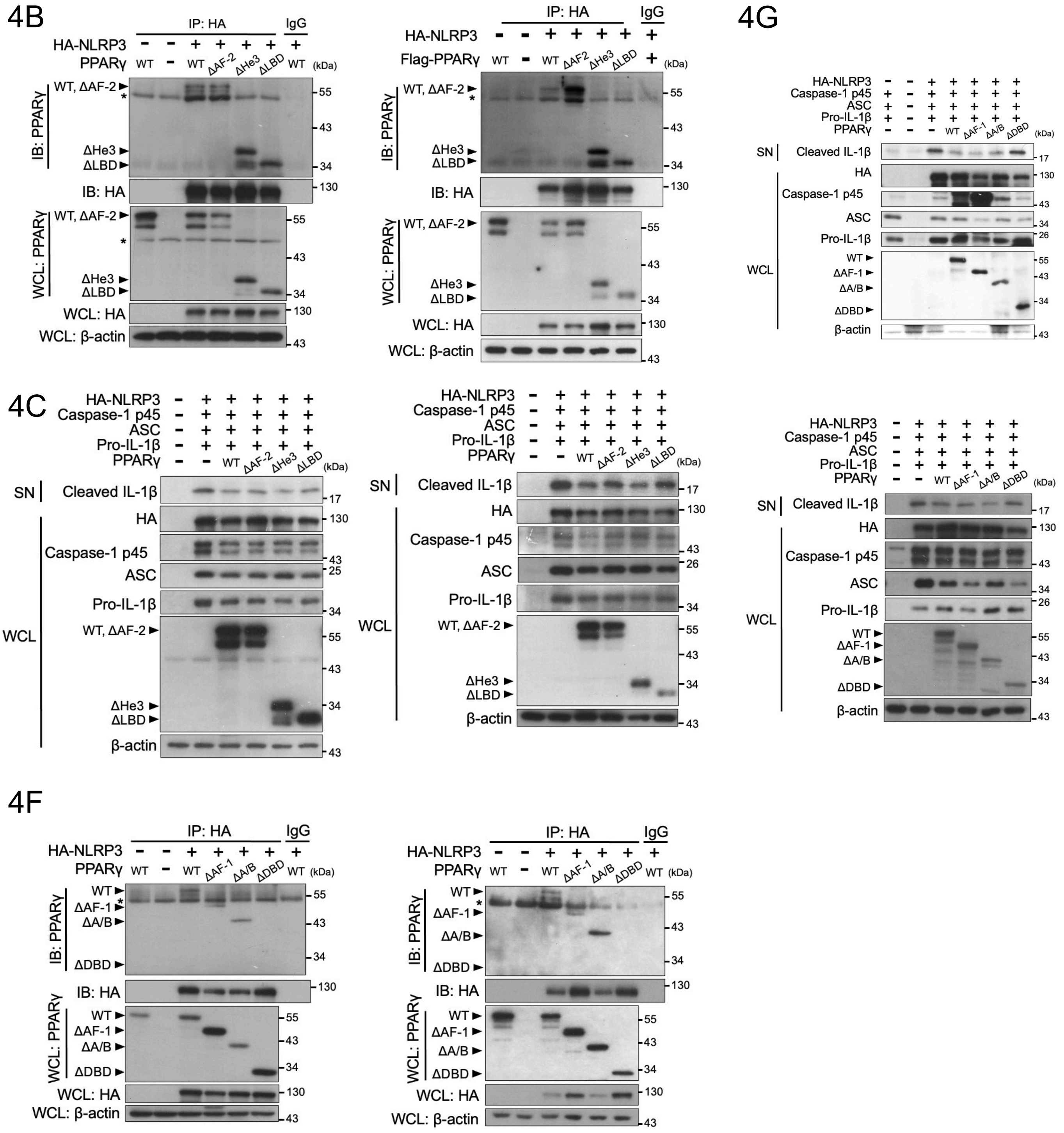

Figure S13. Repeated data of the immunoblotting related to Figure 4.

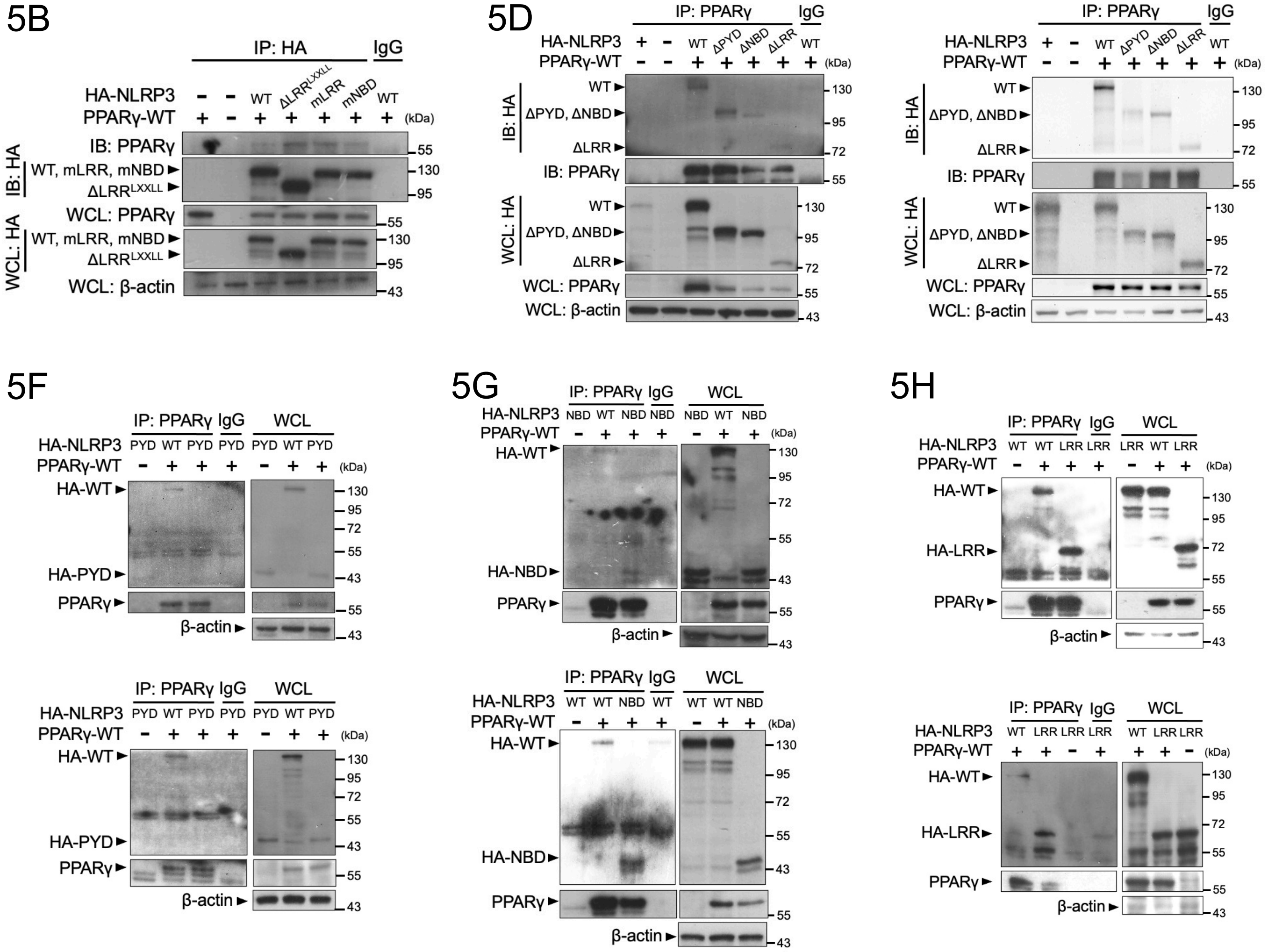

Figure S14. Repeated data of the immunoblotting related to Figure 5.

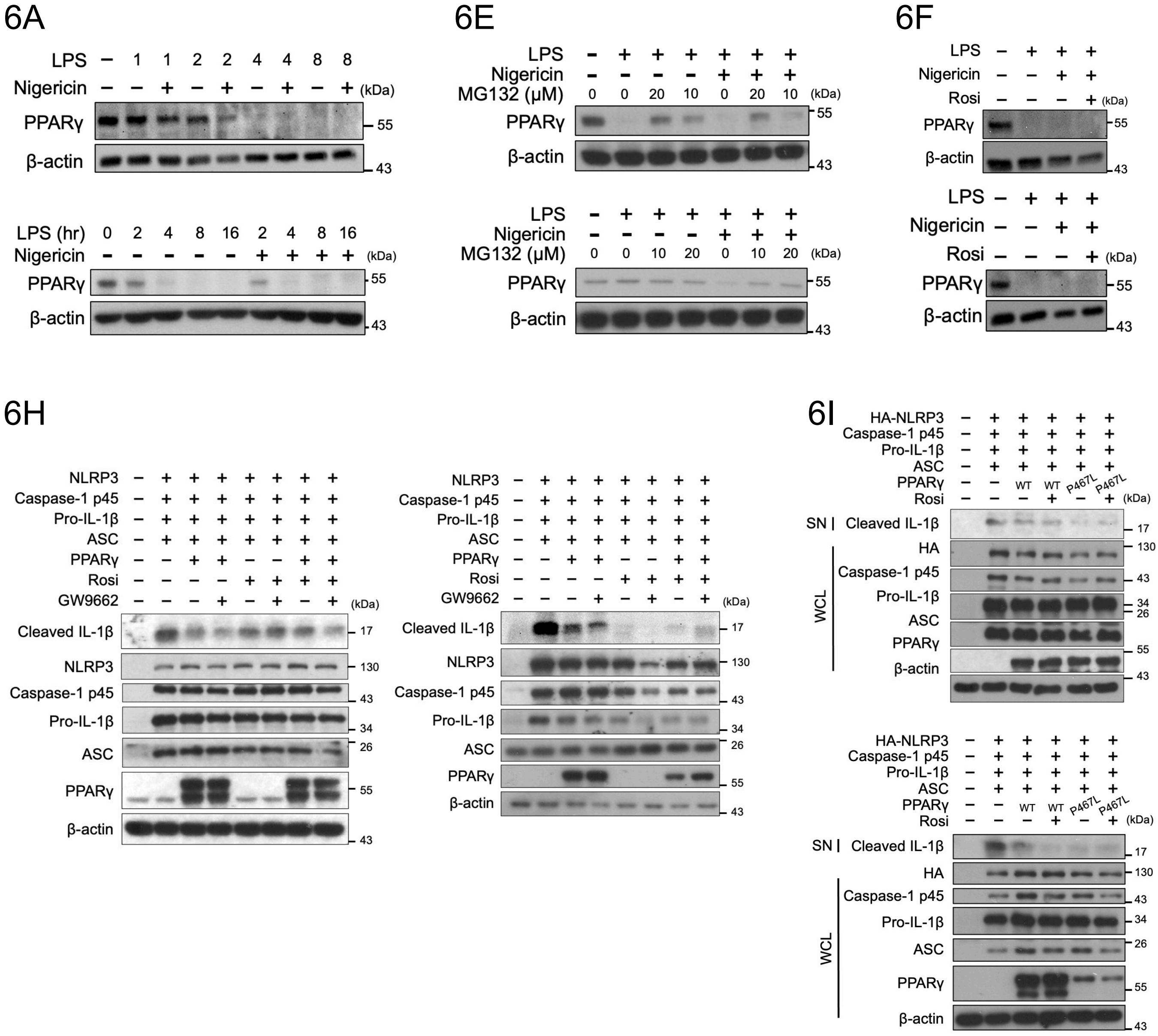

Figure S15. Repeated data of the immunoblotting related to Figure 6.

7A

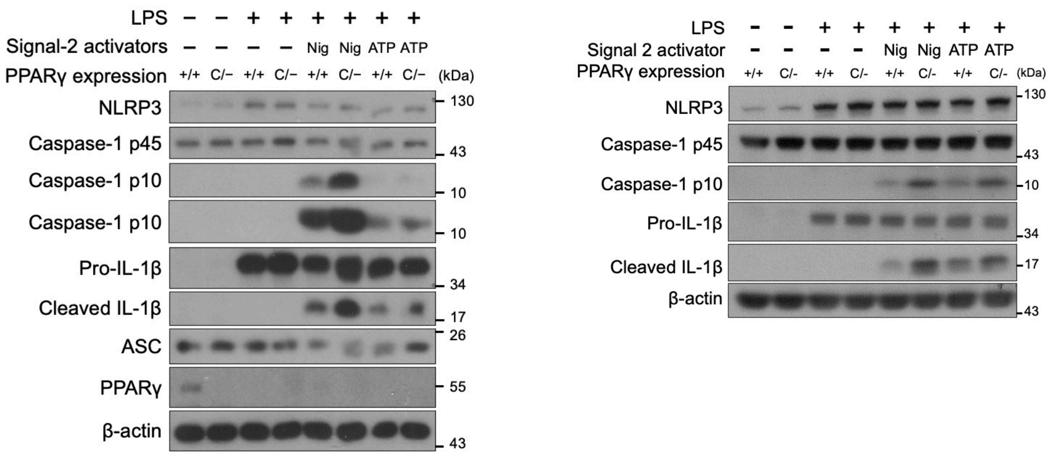

7B

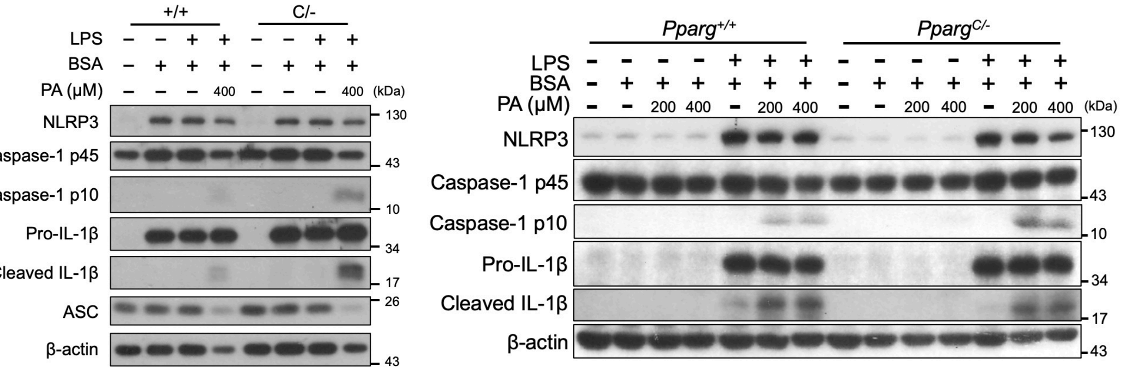

Figure S16. Repeated data of the immunoblotting related to Figure 7.

Supplementary Tables

Table S1. List of plasmids used in experiments.

Plasmid	Vector	Source
HA-NLRP3	pcDNA4	Dr. Ming-Zong Lai, Academia Sinica, Taiwan
ASC		
Pro-caspase-1		
Pro-IL-1β		
PPARγ	pCMV2	Dr. Yau-Sheng Tsai, Institute of Clinical Medicine, National Cheng Kung University, Tainan, Taiwan
PPARγ-ΔAF-2		
PPARγ-ΔHe3		
PPARγ-ΔLBD		
PPARγ-ΔAF-1		
PPARγ-ΔA/B		
PPARγ-ΔDBD		
NLRP3-ΔPYD	pcDNA4	Dr. Yau-Sheng Tsai, Institute of Clinical Medicine, National Cheng Kung University, Tainan, Taiwan
NLRP3-ΔNBD		
NLRP3-ΔLRR		
NLRP3-ΔLRR^{LXXLL}		
NLRP3-mLRR		
NLRP3-mNBD		
NLRP3-PYD		
NLRP3-NBD		
NLRP3-LRR		

Table S2. List of antibodies used in experiments.

Antigen	Host	Cat.	Source	Applications
**Primary antibodies**				
NLRP3	Mouse	AG-20B-0014	Adipogen	WB, IP
	Goat	ab4207	Abcam	IF
PPARγ	Rabbit	#2443	Cell signaling	WB, IP
	Rabbit	sc-7196	Santa Cruz	WB
	Mouse	ab41928	Abcam	IF
ASC	Rabbit	AG-25B-0006	Adipogen	WB, IP, IF
Caspase-1	Rabbit	ab179515	Abcam	WB
IL-1β	Goat	AF-401-NA	R&D systems	WB
c-Myc tag	Mouse	sc-40	Santa Cruz	WB
HA tag	Mouse	MMS-101R	Covance	WB, IP
β-actin	Mouse	A5441	Sigma	WB
Mouse IgG	Mouse	12-371	Millipore	IP
Rabbit IgG	Rabbit	12-370	Millipore	IP
**Secondary antibodies**				
anti-Mouse IgG-HRP	Goat	20102	Leadgene	WB
anti-Rabbit IgG-HRP	Goat	20202	Leadgene	WB
Anti-Goat IgG	Rabbit	5220-0362	Seracare	WB

*WB, western blotting; IP, Immunoprecipitation; IF, Immunofluorescence

Table S3. List of human samples.

S/N	Gender	Age	Procedure type (Mini-gastric bypass, Sleeve gastrectomy, or Gastric banding)	BMI (Before Surgery)	BMI (6mth after Surgery)	BMI (12mth after Surgery)	Involved in panel of Figure 7
1	F	23	Sleeve gastrectomy	43.2	-	30.8	H
2	F	27	Sleeve gastrectomy	41.6	33.5	-	H
3	F	29	Sleeve gastrectomy	42.6	31.4	-	D, F, G
4	F	44	Mini-gastric bypass	48.2	35.5	34.0	D, F, G
5	F	22	Sleeve gastrectomy	41.6	-	-	H
6	F	35	Sleeve gastrectomy	44.1	31.8	-	D
7	F	29	Gastric banding	38.9	32.4	32.0	D, F, G
8	M	39	Sleeve gastrectomy	38.5	27.2	-	D, F, G
9	F	37	Sleeve gastrectomy	48.1	34.5	34.5	D, F, G
10	F	56	Sleeve gastrectomy	34.3	-	-	D
11	F	34	Sleeve gastrectomy	45.6	-	-	D
12	M	27	Sleeve gastrectomy	33.7	-	-	D
13	F	23	Sleeve gastrectomy	37.2	-	-	D
14	F	47	Sleeve gastrectomy	40.5	-	-	H
15	F	57	Sleeve gastrectomy	40.4	-	29.0	H
16	M	28	Sleeve gastrectomy	49.6	-	26.6	H
17	M	40	Sleeve gastrectomy	41.5	29.0	27.9	H
18	M	46	Sleeve gastrectomy	44.1	35	-	H
19	M	32	Mini-gastric bypass	34.2	27	-	H
20	M	25	Control subjects	29.7	-	-	E
21	M	24		34.4	-	-	E
22	M	25		23.3	-	-	E
23	M	24		19.3	-	-	E
24	F	28		25.3	-	-	E
25	F	25		22.0	-	-	E
26	M	35		19.6	-	-	E
27	F	29		27.5	-	-	E
28	F	26		23.3	-	-	E

29	F	25	Control subjects	25.5	-	-	E
30	F	26		18.8	-	-	E
31	F	33		31.2	-	-	E

-: N/A

The subject (S/N: 4) was analyzed in both 6 and 12 months after surgery.

Figure 7G used same subjects with Figure 7F.

1

Table S4. Summary of NBD and LRR sequence analysis between NLRC4 and NLRP3 by BLAST results.

Organism	Domain	Total score	Query coverage	Identity (%)	e-value
Human	LRR	135.0	46%	27.44%	1e-05
	NBD	62.0	63%	21.34%	2e-09
Mouse	LRR	148.0	57%	27.57%	6e-08
	NBD	69.7	65%	24.50%	5e-12
